# Bimetallic Peroxide Nanocomposites‐Driven Redox Dyshomeostasis to Activate Sequential Cuproptosis and Pyroptosis for Amplified Tumor Immunotherapy

**DOI:** 10.1002/advs.202512470

**Published:** 2025-11-23

**Authors:** Guanting He, Haixia Zhu, Peipei Kang, Lili Feng, Chenghao Yu, Yanlin Zhu, Bin Liu, Zhengxiao Guo, Piaoping Yang

**Affiliations:** ^1^ Key Laboratory of Superlight Materials and Surface Technology Ministry of Education College of Materials Science and Chemical Engineering Harbin Engineering University Harbin 150001 P. R. China; ^2^ Cancer Institute Affiliated Tumor Hospital of Nantong University Nantong 226631 P. R. China; ^3^ Department of Chemistry The University of Hong Kong Pokfulam Hong Kong (SAR) 999077 China

**Keywords:** anti‐tumor immunotherapy, bimetallic peroxide, cuproptosis, immunogenic cell death, pyroptosis

## Abstract

Although metal peroxides are extensively employed in tumor therapy, novel synergistic tumor treatment approaches based on the combination of multiple types of metal peroxides are still lacking and warrant further exploration. To overcome this challenge, hyaluronic acid (HA)‐modified bimetallic peroxide nanocomposites (MgO_2_‐CuO_2_@HA NCs) are developed by combining magnesium peroxide (MgO_2_) nanosheets and short‐grained copper peroxide (CuO_2_) nanodots. By modifying HA to enhance tumor targeting and stability, MgO_2_‐CuO_2_@HA NCs leverage pH‐dependent decomposition to release Mg^2+^, H_2_O_2_, and Cu^2+^ under acidic conditions, thereby initiating Fenton‐like reactions for the generation of hydroxyl radicals (•OH), while simultaneously depleting glutathione to generate Cu^+^. This process induces cuproptosis through the Cu^+^‐mediated oligoaggregation of dihydrolipoamide S‐acetyltransferase. Additionally, enhanced •OH activates pyroptosis via the caspase‐1/gasdermin D pathway. Cuproptosis and pyroptosis can induce immunogenic cell death, thereby triggering the anti‐tumor immune responses. Notably, released Mg^2+^ can enhance the activation of CD8^+^ T cells by promoting the conformational activation of leukocyte function‐associated antigen 1. Therefore, this study establishes a novel paradigm for synergistic anti‐tumor immunotherapy based on bimetallic peroxide nanocomposites, offering promising prospects for clinical immunotherapy.

## Graphical abstract

Hyaluronic acid‐modified bimetallic peroxide nanocomposites (MgO_2_‐CuO_2_@HA) are designed for synergistic tumor therapy. The nanocomposites release Mg^2+^, H_2_O_2_, and Cu^2+^ in tumor cells, induce cuproptosis via Cu^+^‐mediated protein aggregation, and activate pyroptosis through caspase‐1/gasdermin D pathways for inducing immunogenic cell death, collectively promote the activation of CD8^+^ T cells by released Mg^2+^ for cancer immunotherapy with minimal systemic toxicity.

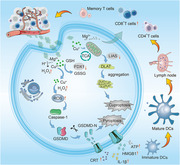

## 1 Introduction

Cancer is one of the most lethal diseases, and cancer poses a significant threat to human health and life. Although various treatment strategies have been developed for clinical applications, such as radiotherapy and chemotherapy, the high side effects and limited efficacy of these therapies remain critical challenges that need to be addressed.^[^ ^1^, ^2^ ^]^ Immunotherapy is an innovative approach that leverages the immune system of patients to combat tumors.^[^ ^3^, ^4^, ^5^ ^]^ In many tumor immunotherapy processes, the introduction and delivery of tumor antigens play a vital role.^[^ ^6^, ^7^ ^]^ The nano‐vaccines derived from tumor cells do not require cumbersome steps of new antigen screening and synthesis. Moreover, they can offer all potential antigens to generate various immune responses, avoiding the issue of tumor antigen loss that may arise in the new antigen strategy. Although this “ex vivo” tumor cell vaccine has a strong anti‐tumor immune ability, the manufacturing process is complex and time‐consuming.^[^ ^8^, ^9^, ^10^, ^11^ ^]^ Inducing immunogenic cell death (ICD) is a promising strategy for enhancing immunotherapy sensitivity. ICD triggers the adaptive immune response by releasing damage‐associated molecular patterns (DAMPs), including high mobility group box 1 protein (HMGB1), calreticulin (CRT), and adenosine triphosphate (ATP), which collectively facilitate dendritic cell maturation and cytotoxic T lymphocyte activation, converting the entire tumor cells into “in situ” cancer vaccines.^[^ ^12^, ^13^, ^14^ ^]^ However, the most effective ICD is achieved by inducing apoptosis, which often yields unsatisfactory therapeutic outcomes owing to the intrinsic tumor resistance to apoptosis.^[^ ^15^, ^16^, ^17^, ^18^ ^]^ Consequently, there is an urgent need to explore novel modalities for cancer cell death with enhanced immunogenicity, particularly by exploring alternative cell death pathways such as ferroptosis, cuproptosis, and pyroptosis. Such approaches may overcome the tumor immune escape mechanism while establishing a durable anti‐tumor immune memory. For instance, Dai et al. employed amphiphilic and polyphenolized semiconductor polymers as carriers, which can self‐assemble and encapsulate ferroptosis inducer (Fe^3+^) and exosome inhibitor (GW4869) through the coordination of metal polyphenols, thereby constructing a phototheranostic metal–polyphenol network. This novel phototheranostic metal–polyphenol network realizes immune reinforcement, effectively stimulates anti‐tumor immune effects, and persistently maintains specific immune memory.^[^ ^19^ ^]^

Cuproptosis is a copper (Cu)‐dependent programmed cell death pathway, characterized by mitochondrial dysfunction triggered by intracellular Cu accumulation.^[^ ^20^, ^21^, ^22^, ^23^ ^]^ Excessive Cu can promote the aggregation of acylated mitochondrial enzymes and destabilize iron–sulfur (Fe–S) cluster proteins, thus disrupting cellular metabolic homeostasis. As an essential trace element, Cu serves as a cofactor for enzymes involved in critical processes such as oxidative phosphorylation, redox balance, and macromolecule synthesis. Although Cu homeostasis is strictly regulated by conserved transporters and chaperone proteins, the pathological copper accumulation of Cu can circumvent these protective mechanisms and trigger cuproptosis. Importantly, this pathway is independent of the classical apoptotic signaling cascade and offers a novel strategy for combating apoptosis‐resistant malignant tumors. Zhang et al. developed Cu‐based metal–organic framework nanoparticles and modified them with *Rhodobacter sphaeroides*‐derived bacterial membrane vesicles for photothermal effects and cuproptosis‐mediated ICD, thereby boosting the anti‐tumor immune response.^[^ ^24^ ^]^ Moreover, recent investigations have also revealed that Cu ions exhibit a potent Fenton‐like reaction that generates reactive oxygen species (ROS) by catalyzing the excessive hydrogen peroxide (H_2_O_2_) within tumor cells.^[^ ^25^ ^]^

Pyroptosis is a form of inflammatory programmed cell death characterized by inflammasome activation, caspase‐1‐dependent gasdermin D (GSDMD) cleavage, and the perforation of plasma membrane perforation.^[^ ^26^, ^27^, ^28^ ^]^ Mechanistically, the inflammasome complexes sense cellular stress or infection and trigger the activation of caspase‐1. This activation process facilitates the cleavage of GSDMD, releasing its N‐terminal pore‐forming domain (GSDMD‐N) to initiate pyroptosis. These pores promote cell swelling, membrane rupture, and the release of pro‐inflammatory cytokines (e.g., interleukin (IL)‐18 and IL‐1β) and tumor antigens. ROS plays a dual role in pyroptosis by directly inducing membrane damage and amplifying inflammatory signals.^[^ ^29^, ^30^ ^]^ Notably, the elevated intracellular levels of metal ions (e.g., Cu^2+^ and Fe^3+^) can generate ROS through Fenton‐like reactions, further disrupting the redox homeostasis and exacerbating pyroptotic cell death.^[^ ^31^, ^32^, ^33^, ^34^, ^35^ ^]^ In addition, many studies have indicated that the excessive metal ions (such as K^+^, Na^+^, Zn^2+^, and Ca^2+^) within tumor cells can also lead to pyroptosis of tumor cells.^[^ ^36^, ^37^, ^38^, ^39^, ^40^ ^]^ Pyroptosis has emerged as a promising strategy in cancer therapy because it can eliminate tumor cells and simultaneously stimulate anti‐tumor immune responses.^[^ ^41^, ^42^, ^43^ ^]^ However, the balance between pyroptosis and inflammatory toxicity remains a challenge. Targeted modulation of pyroptotic pathways, particularly via ROS amplification and metal ion regulation, represents a frontier in precision immunotherapy for harnessing both tumor eradication and immune activation.

Compared to normal cells, the abnormal metabolism in tumor cells causes a hypoxic and acidic microenvironment.^[^ ^44^, ^45^ ^]^ H_2_O_2_ content in tumor cells is slightly high; however, this limited concentration for inducing the ROS generation is not ideal.^[^ ^46^, ^47^ ^]^ To further elevate the intracellular H_2_O_2_ levels within tumor cells to facilitate the generation of more ROS and induce tumor cell death, multiple methodologies have emerged, such as direct transportation or catalytic conversion by glucose oxidase.^[^ ^48^, ^49^, ^50^ ^]^ Nevertheless, these approaches frequently yield suboptimal outcomes owing to constraints such as low‐drug loading efficiency and a hypoxic tumor microenvironment. Owing to their distinct reactive properties and the ability to release metal ions and H_2_O_2_ from tumor cells, which secrete substantial amounts of lactic acid, metal peroxide nanoparticles have attracted considerable attention.^[^ ^51^, ^52^, ^53^ ^]^ Therefore, the responsive H_2_O_2_ compensation properties of metal peroxides can effectively promote ROS generation within tumor cells. However, the effect of single‐metal peroxide nanoparticles in generating ROS is limited, and they must be used in combination with other components. It is imperative to highlight that the impact of metal peroxide nanoparticles on biosafety merits significant attention. Consequently, the development of bimetallic peroxide nanoparticles that can undergo responsive decomposition at the tumor sites and cause the multi‐pathway death of tumor cells is of great significance for improving the effectiveness of tumor immunotherapy.^[^ ^54^, ^55^, ^56^ ^]^

**Scheme 1:**
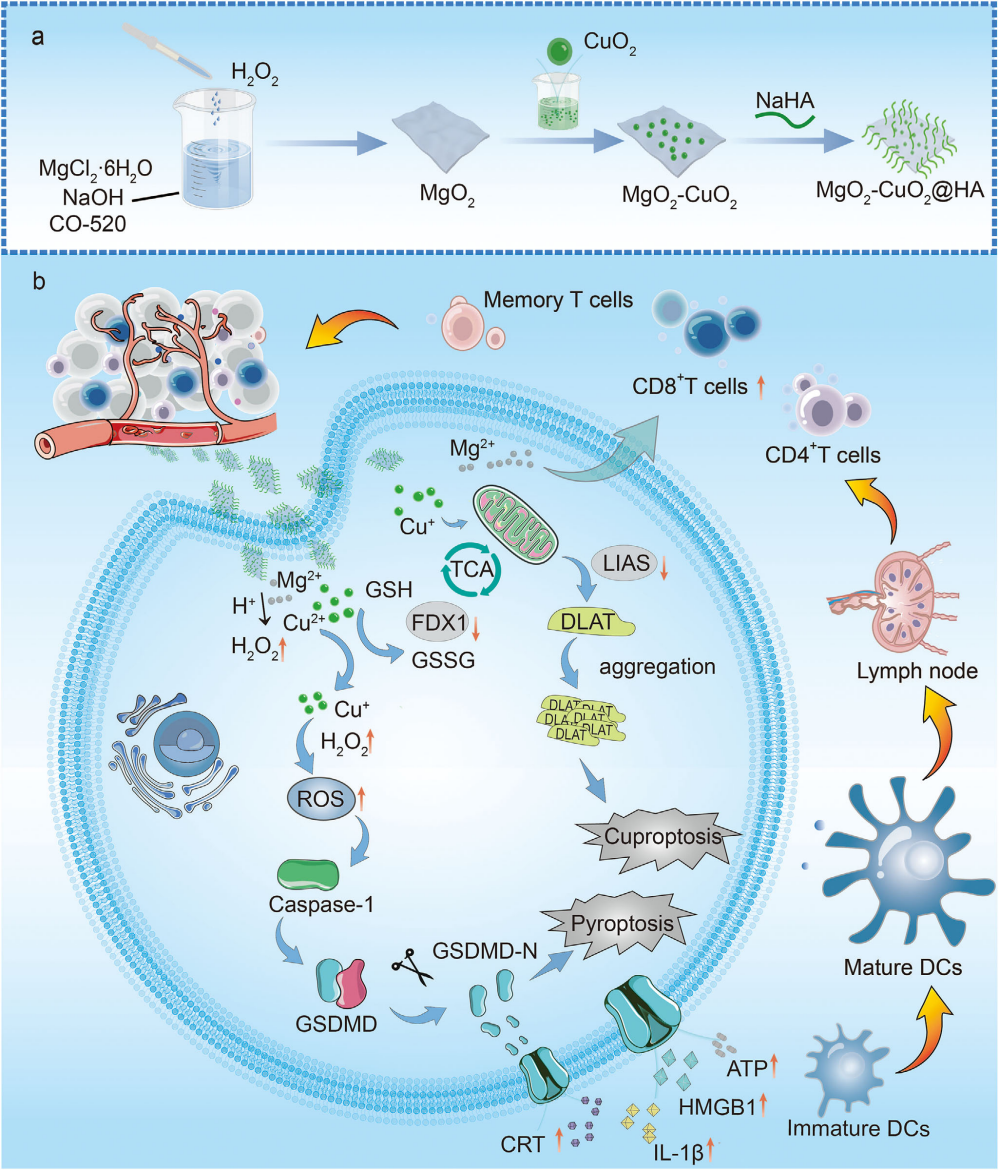
a) Schematic representation of the fabrication process of the MgO_2_‐CuO_2_@HA NCs. b) Anti‐tumor immunotherapy mechanism of MgO_2_‐CuO_2_@HA NCs induced by cuproptosis and pyroptosis.

Herein, we synthesized nanocomposites of magnesium peroxide (MgO_2_) nanosheets and copper peroxide (CuO_2_) nanodots modified with hyaluronic acid (HA) (MgO_2_‐CuO_2_@HA NCs) by coordinating Cu^2+^ and Mg^2+^ with hydroxide ions at room temperature, facilitated by H_2_O_2_, as shown in **Scheme** **1**a. Owing to the excellent biocompatibility and targeting properties of HA, MgO_2_‐CuO_2_@HA NCs demonstrated significant accumulation at the tumor sites. Following phagocytosis by tumor cells, Cu^2+^, Mg^2+^, and H_2_O_2_ were rapidly released. Cu^2+^ catalyzed the self‐supply of H_2_O_2_ via a Fenton‐like reaction, generating abundant •OH while depleting glutathione (GSH) to produce Cu^+^. Cuproptosis occurred via Cu^+^‐mediated oligomerization of dihydrolipoamide S‐acetyltransferase (DLAT) and consumption of Fe‐S cluster proteins. Pyroptosis was triggered by the bursting of metal ions and enhanced ROS production via the caspase‐1/GSDMD pathway. ICD, induced by cuproptosis and pyroptosis, releases a substantial amount of DAMPs, effectively stimulating the host immune response (Scheme 1b). The released Mg^2+^ promotes the conformational activation of leukocyte function‐associated antigen 1 (LFA‐1) on CD8^+^ T cells by binding to the metal‐ion‐dependent adhesion site, thereby enhancing the activation of CD8^+^ T cells.^[^ ^57^, ^58^ ^]^ Overall, MgO_2_‐CuO_2_@HA NCs can significantly suppress tumor cell growth through the synergistic effects of cuproptosis, pyroptosis, and induced immune responses, offering promising prospects for clinical immunotherapy.

## 2 Results and Discussion

**Figure 1:**
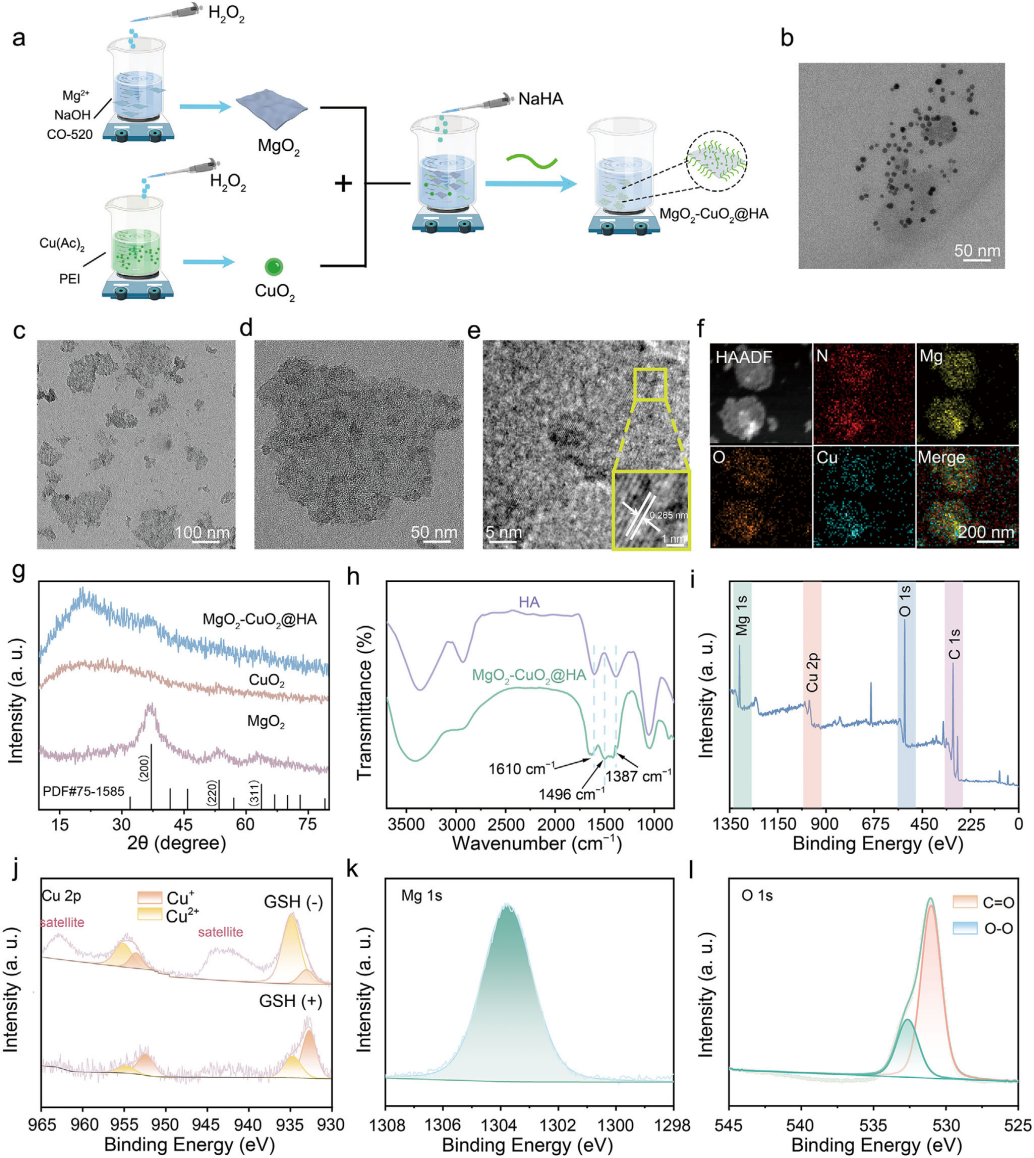
Structural and compositional characterization. a) Schematic illustration of the synthetic procedure for MgO_2_‐CuO_2_@HA NCs. TEM images of b) CuO_2_, c) MgO_2_, and d) MgO_2_‐CuO_2_@HA NCs. e) HRTEM image of MgO_2_‐CuO_2_@HA NCs. f) The corresponding HAADF‐STEM and elemental mapping images for MgO_2_‐CuO_2_@HA NCs. g) XRD patterns of different samples. h) FT‐IR spectra of HA and MgO_2_‐CuO_2_@HA NCs. i) XPS survey spectrum of MgO_2_‐CuO_2_@HA NCs. j) XPS high‐resolution spectra of Cu 2p for MgO_2_‐CuO_2_@HA NCs reacted without or with GSH. XPS high‐resolution spectra of k) Mg 1s and l) O 1s for MgO_2_‐CuO_2_@HA NCs.

The synthesis of MgO_2_‐CuO_2_@HA NCs involved a straightforward combination of pre‐synthesized CuO_2_ nanodots with MgO_2_ nanosheets, followed by stirring in an aqueous sodium hyaluronate solution at ambient temperature, as outlined in **Figure** **1**a. Transmission electron microscopy (TEM) images reveal the successful fabrication of CuO_2_ nanodots with an average size of ≈5 nm (Figure 1b). MgO_2_ nanosheets were synthesized using the reverse‐phase microemulsion method, and exhibited a flake structure with a diameter of ≈150 nm (Figure 1c), which was highly analogous to the morphology of MgO_2_‐CuO_2_@HA NCs after combination with CuO_2_ nanodots (Figure 1d). According to the statistical analysis of the TEM image (Figure S1, Supporting Information), the average size of MgO_2_‐CuO_2_@HA NCs was 150 nm. During the synthesis of MgO_2_‐CuO_2_ NCs, the dosage of MgO_2_ nanosheets was kept constant, while the addition ratio of CuO_2_ nanodots was precisely adjusted to fabricate a series of composite materials. Subsequently, a systematic evaluation was conducted on the release behavior of Cu^2+^ from these MgO_2_‐CuO_2_ NCs after acid treatment (pH = 5.5). As the amount of added CuO_2_ nanodots increased, the concentration of Cu^2+^ released from the MgO_2_‐CuO_2_ NCs exhibited an upward trend. Notably, when the mass ratio of MgO_2_ to CuO_2_ decreased below 1:3, the release of Cu^2+^ tended to stabilize without significant changes (Figure S2, Supporting Information). These findings suggest that the optimal mass ratio of MgO_2_ to CuO_2_ was 1:3. At this specific ratio, the surface of the MgO_2_ nanosheets enabled the efficient loading and homogeneous distribution of the CuO_2_ nanodots. Additionally, the hydrodynamic sizes of MgO_2_‐CuO_2_@HA NCs in various solvents were evaluated, as shown in Figure S3 (Supporting Information). During the 7‐day cultivation process, MgO_2_‐CuO_2_@HA NCs exhibited stable hydrated particle sizes and relative polydispersity Index values, indicating good stability under physiological conditions. The high‐resolution TEM image reveals that the lattice spacing of the as‐prepared MgO_2_‐CuO_2_@HA NCs was 0.265 nm (Figure 1e). Elemental mapping analysis (Figure 1f) confirmed the uniform distribution of N, O, Mg, and Cu within MgO_2_‐CuO_2_@HA NCs. As displayed in Figure 1g, the X‐ray diffraction pattern of the MgO_2_‐CuO_2_@HA NCs was matched well with that of MgO_2_ nanosheets, due to the presence of amorphous CuO_2_ nanodots. The Fourier transform infrared spectra revealed the structural composition of MgO_2_‐CuO_2_@HA NCs. As shown in Figure 1h, during the reaction process of MgO_2_‐CuO_2_@HA NCs, the carboxylate groups (─COO^−^) of HA acted as Lewis bases and coordinated directly with the Lewis acidic metal centers on the surface of MgO_2_‐CuO_2_ NCs. The characteristic peaks at 1610 and 1387 cm^−1^ corresponded to the ν_CO_ vibration of the amide and carboxyl groups in HA. These characteristic signal peaks remained in the spectrum of MgO_2_‐CuO_2_@HA NCs. In addition, another peak at 1496 cm^−1^ was detected, pointing out the formation of five‐ and six‐membered ring structures via the interaction between the four hydroxyl groups in each HA unit and the MgO_2_‐CuO_2_ component. This observation indicates that HA was firmly anchored to the surface of the MgO_2_‐CuO_2_ NCs. Thermal gravimetric analysis curves indicated that the weight percentage of HA on the surface of MgO_2_@HA was ≈15.28%, whereas that on the surface of CuO_2_@HA was ≈14.57%. Furthermore, the weight percentage of HA on the surface of MgO_2_‐CuO_2_@HA NCs was ≈16.8%. These findings confirm the successful surface modification and similar weight percentages of HA on the surfaces of the three NCs (Figure S4, Supporting Information). As shown in Figure S5 (Supporting Information), due to the different charge characteristics on the surfaces of MgO_2_ nanosheets and CuO_2_ nanodots, the CuO_2_ nanodots were attracted by the electrostatic forces and deposited on the surface of the MgO_2_ nanosheets, and the zeta potential of MgO_2_‐CuO_2_@HA NCs was transformed from −6.01 to −15.86 mV after the surface modification with HA. Atomic force microscopy (AFM) was employed to effectively characterize the successful incorporation of CuO_2_ nanodots onto the MgO_2_ nanosheets. Additionally, the AFM image and corresponding height profile of the MgO_2_ nanosheets reveal an average thickness of 32 nm (Figure S6, Supporting Information). Following the loading of the CuO_2_ nanodots, the AFM image of the MgO_2_‐CuO_2_@HA NCs and their corresponding height profiles demonstrate that the average thickness increased to 40 nm (Figure S7, Supporting Information). This increase in thickness confirmed the successful integration of the CuO_2_ nanodots onto the MgO_2_ nanosheets. Energy‐dispersive X‐ray spectroscopy and X‐ray photoelectron spectroscopy (XPS) were used to analyze the chemical composition of MgO_2_‐CuO_2_@HA NCs (Figure 1i; Figure S8, Supporting Information). The XPS survey spectrum showed that MgO_2_‐CuO_2_@HA NCs were mainly composed of Mg, Cu, O, and C. In Figure 1j–l, the high‐resolution XPS profiles of Cu 2p, Mg 1s, and O 1s are presented. The high‐resolution XPS spectrum of Cu 2p exhibits four prominent peaks at 934.9 eV (Cu 2p_3/2_) and 955.2 eV (Cu 2p_1/2_), which were characteristic of Cu^2+^ species, and at 933.1 eV (Cu 2p_3/2_) and 953.5 eV (Cu 2p_1/2_), which were assigned to Cu^+^ species (Figure 1j). After incubation with GSH, the characteristic peaks of the Cu^2+^ species were markedly attenuated, whereas the peaks associated with the Cu^+^ species became more prominent. This change was primarily attributed to the reduction of Cu^2+^ to Cu^+^ mediated by GSH. The high‐resolution XPS spectrum of Mg 1s shows a single peak at 1303.7 eV (Figure 1k). Furthermore, the O 1s spectrum displays two characteristic peaks at 531.3 and 533 eV, which were related to C═O and O─O bonds respectively, indicating the presence of HA and peroxy groups in MgO_2_‐CuO_2_@HA NCs (Figure 1l). These comprehensive characteristics confirm the successful fabrication of MgO_2_‐CuO_2_@HA NCs with preserved peroxide functionality and redox‐active Cu species.

**Figure 2:**
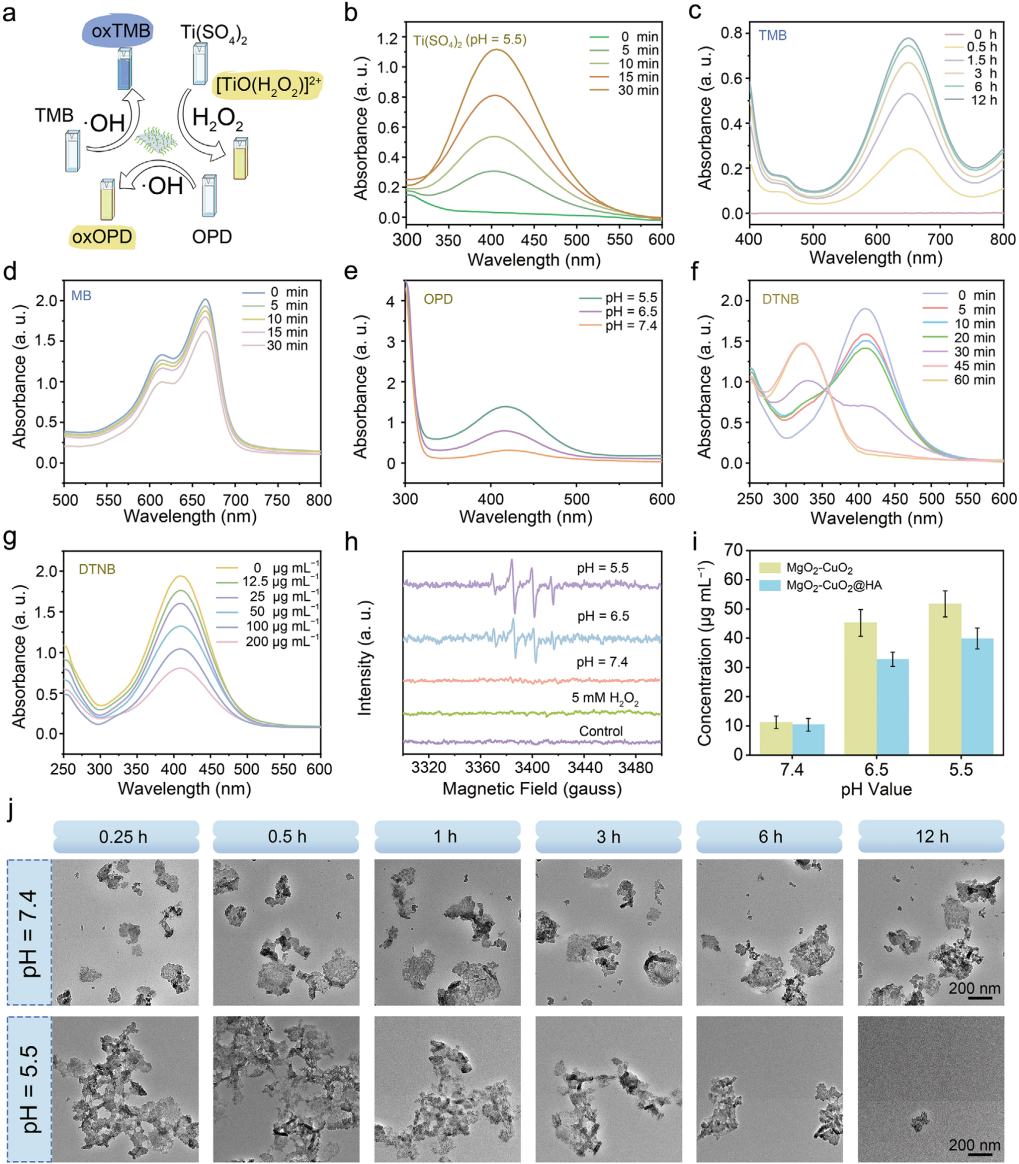
a) Schematic depiction of the catalytic action of MgO_2_‐CuO_2_@HA NCs. b) UV–vis absorption spectra of Ti(SO_4_)_2_ solution incubated with MgO_2_‐CuO_2_@HA NCs for different time intervals. c,d) The oxidation of TMB and degradation of MB mediated by •OH produced from MgO_2_‐CuO_2_@HA NCs for various reaction times at pH = 5.5. e) UV–vis absorption spectra of OPD solution catalyzed by MgO_2_‐CuO_2_@HA NCs solution with different conditions. f) The time‐dependent consumption of GSH by MgO_2_‐CuO_2_@HA NCs. g) The analysis of GSH depletion by MgO_2_‐CuO_2_@HA NCs with varying concentrations. h) ESR spectra of DMPO‐•OH adducts generated by MgO_2_‐CuO_2_@HA NCs at different conditions. i) Cu^2+^ release from MgO_2_‐CuO_2_ and MgO_2_‐CuO_2_@HA NCs dispersed in solution with different pH values (*n* = 5, mean ± S.D.). j) TEM images of MgO_2_‐CuO_2_@HA NCs exposed to acidic (pH = 5.5) and neutral (pH = 7.4) environments for various incubation time periods.

In general, the titanium sulfate (Ti(SO_4_)_2_) method was employed to evaluate the capacity of MgO_2_‐CuO_2_@HA NCs to generate H_2_O_2_ under slightly acidic conditions. The ability of MgO_2_‐CuO_2_@HA NCs to produce •OH was investigated using o‐phenylenediamine (OPD) and 3,3′,5,5′‐tetramethylbenzidine (TMB) assays, in which the Cu^2+^ could catalyze the decomposition of H_2_O_2_ through Fenton‐like reaction (**Figure** **2**a). To detect the production of H_2_O_2_, the absorbance of the Ti(SO_4_)_2_ solution was measured at 410 nm under different conditions. As shown in Figure 2b and Figure S9 (Supporting Information), the absorbance of Ti(SO_4_)_2_ exhibited the most significant time‐dependent change after treatment under weakly acidic conditions (pH = 5.5), suggesting that the H_2_O_2_ production capacity of MgO_2_‐CuO_2_@HA NCs was influenced by solution acidity. In addition, the amount of H_2_O_2_ generated from MgO_2_‐CuO_2_@HA NCs was significantly higher than that generated from the other groups (Figure S10, Supporting Information). The influence of the different reaction conditions on the oxidation rate of TMB was also investigated (Figure 2c; Figure S11, Supporting Information). Compared with the other groups, MgO_2_‐CuO_2_@HA NCs demonstrated the strongest •OH generation capability under mildly acidic conditions (pH = 5.5). The generation of •OH was also confirmed by monitoring the degradation of methyl blue (MB). As shown in Figure 2d, after treatment with MgO_2_‐CuO_2_@HA NCs under mildly acidic conditions, the absorbance value of MB at 664 nm decreased significantly over the incubation period, which proved the ability of MgO_2_‐CuO_2_@HA NCs to continuously generate •OH. To further evaluate •OH generation, OPD was employed as a probe, because its oxidation by •OH yielded a characteristic absorption peak at 417 nm. As depicted in Figure 2e and Figure S12 (Supporting Information), the production of •OH by MgO_2_‐CuO_2_@HA NCs also displayed time and acidity‐dependent increases. The enhanced oxidation activity can be attributed to the self‐supply of H_2_O_2_ by MgO_2_‐CuO_2_@HA NCs under slightly acidic conditions, which in turn accelerated the Fenton‐like reaction involving Cu^2+^, thereby producing a substantial amount of •OH.

The GSH consumption capacity of MgO_2_‐CuO_2_@HA NCs was evaluated. 5,5‐dithiobis(2‐nitrobenzoic acid) (DTNB) serves as a colorimetric marker for GSH detection, and forms yellow compounds by interacting with the ─SH group of GSH. When DTNB was added to the mixture of MgO_2_‐CuO_2_@HA NCs and GSH, the absorbance of the yellow product at 412 nm gradually diminished as the incubation time prolonged and concentration increased, indicating the GSH depletion caused by MgO_2_‐CuO_2_@HA NCs (Figure 2f,g). The strong capability of MgO_2_‐CuO_2_@HA NCs in GSH consumption is beneficial for reducing ROS quenching. Furthermore, using 5,5‐dimethyl‐1‐pyrroline‐N‐oxide (DMPO) as a spin‐trapping agent in electron spin resonance (ESR) spectroscopy analysis, it was found that MgO_2_‐CuO_2_@HA NCs could initiate the production of •OH, as shown in Figure 2h. Compared with the other groups, the MgO_2_‐CuO_2_@HA NCs treated with a mildly acidic solution (pH = 5.5) exhibited more distinct signal peaks of DMPO/•OH with a relative intensity ratio of 1:2:2:1. We then verified the •OH generation abilities of different nanomaterials under the same conditions. Compared with the MgO_2_ nanosheets and CuO_2_ nanodots, MgO_2_‐CuO_2_@HA NCs group exhibited the strongest ability to produce •OH (Figure S13, Supporting Information).

The acid‐triggered breakdown mechanism of MgO_2_‐CuO_2_@HA NCs was thoroughly examined to investigate their pH‐responsive decomposition behavior. The release of Mg^2+^ and Cu^2+^ from MgO_2_‐CuO_2_@HA NCs was quantitatively measured in different buffer solutions with varying pH values using inductively coupled plasma mass spectrometry. The amount of metal ions released was closely related to the pH value. As shown in Figure 2i and Figure S14 (Supporting Information), a negligible release of Mg^2+^ and Cu^2+^ occurred under neutral conditions (pH = 7.4), indicating minimal decomposition degree at physiological pH levels. Nevertheless, as the pH decreased to 6.5 and 5.5, a more significant increase in the release of both metal ions was observed, further confirming the pH‐dependent degradation capability of MgO_2_‐CuO_2_@HA NCs. Under the same treatment conditions, the release of Mg^2+^ and Cu^2+^ from MgO_2_‐CuO_2_@HA NCs was lower than that from MgO_2_‐CuO_2_ NCs, indicating that HA surface modification reduced the direct interaction between the MgO_2_‐CuO_2_ NCs and the surrounding solution. A TEM image analysis was conducted to visually assess the structural integrity of MgO_2_‐CuO_2_@HA NCs under different pH conditions, and the results are shown in Figure 2j. The results revealed that the MgO_2_‐CuO_2_@HA NCs exhibited visible structural decomposition with increasing incubation time (from 0.25 to 12 h) in mildly acidic media, which was consistent with the ion release results. In contrast, the morphological integrity of MgO_2_‐CuO_2_@HA NCs remained stable in neutral environments. These results collectively emphasize the strong pH‐responsive characteristics of MgO_2_‐CuO_2_@HA NCs, which are critical for their potential applications in pH‐triggered drug delivery systems and other stimuli‐responsive nanotechnology platforms.

**Figure 3:**
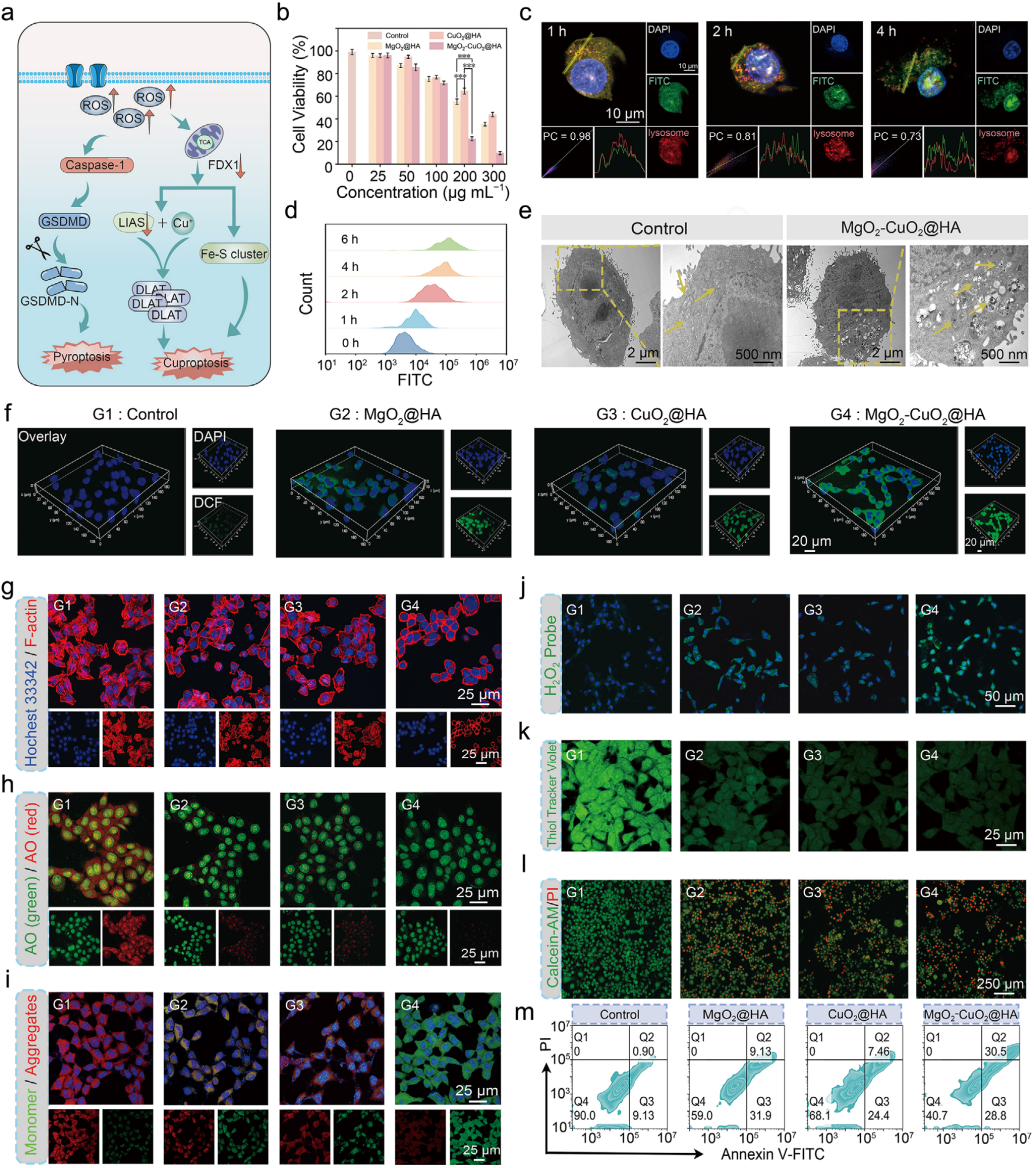
a) Schematic illustration of the cooperative activation of pyroptosis and cuproptosis triggered by MgO_2_‐CuO_2_@HA NCs. b) Cell viability of 4T1 cells with different treatments (*n* = 5, mean ± S.D.). c) Representative CLSM images showed the colocalization of FITC‐labeled MgO_2_‐CuO_2_@HA NCs with the lysosome in 4T1 cells with different incubation times. d) The time‐dependent cellular uptake of FITC‐modified MgO_2_‐CuO_2_@HA NCs in 4T1 cells was quantified by flow cytometry. e) Bio‐TEM images of 4T1 cells incubated with or without MgO_2_‐CuO_2_@HA NCs (200 µg mL^−1^) for 4 h. f) Intracellular ROS levels after treated with various formulations. g) F‐actin cytoskeletal morphology, h) acridine orange staining, i) JC‐1 staining, j) intracellular H_2_O_2_ detection, and k) GSH depletion of 4T1 cells treated with various formulations. l) Fluorescent images of 4T1 cells labeled with Calcein‐AM/PI and m) the cell apoptosis rates of 4T1 cells with different treatments determined by flow cytometry analysis. The groups were divided into (G1) Control, (G2) MgO_2_@HA, (G3) CuO_2_@HA, (G4) MgO_2_‐CuO_2_@HA NCs. Statistical significance was calculated using Student′s *t‐*test: *p < 0.05, **p < 0.01, and ***p < 0.001.

To further assess the in vitro anti‐tumor therapeutic efficacy of MgO_2_‐CuO_2_@HA NCs (**Figure** **3**a), we first assessed their cytotoxicity in 4T1 cells by using the methylthiazolyldiphenyl‐tetrazolium bromide (MTT) assay. Specifically, 4T1 cells were co‐incubated with varying concentrations of MgO_2_@HA nanosheets, CuO_2_@HA nanodots, and MgO_2_‐CuO_2_@HA NCs. As illustrated in Figure 3b, the viability of 4T1 cells treated with MgO_2_‐CuO_2_@HA NCs exhibited a pronounced concentration‐dependent decrease. Notably, the decline in cell viability was significantly greater than that in cells treated with either MgO_2_@HA nanosheets or CuO_2_@HA nanodots alone, suggesting that MgO_2_‐CuO_2_@HA NCs had a superior inhibitory effect on tumor cells. However, MgO_2_‐CuO_2_@HA NCs presented negligible cytotoxicity toward L929 cells within the concentration range of 0–300 µg mL^−1^ (Figure S15, Supporting Information). To examine the internalization mechanism of MgO_2_‐CuO_2_@HA NCs by cancer cells, 4T1 cells were treated with fluorescein isothiocyanate (FITC)‐labeled MgO_2_‐CuO_2_@HA NCs for different time intervals. Subsequently, the cells were analyzed by confocal laser scanning microscopy (CLSM) (Figure 3c). The results demonstrated that the fluorescence intensity of cells treated with FITC‐labeled MgO_2_‐CuO_2_@HA NCs was significantly increased with prolonged incubation time, indicating effective cellular uptake of the nanocomposites. As shown in Figure 3d and Figure S16 (Supporting Information), the flow cytometry results indicated that MgO_2_‐CuO_2_@HA NCs exhibited a more distinct cellular uptake efficiency than that of MgO_2_‐CuO_2_ NCs because of the tumor‐targeting capability conferred by HA modification. Rhodamine B hydrazide (RBH) was used to measure the intracellular Cu^2+^ levels. CLSM images show that the cells treated with MgO_2_‐CuO_2_@HA NCs exhibited the highest Cu^2+^ accumulation (Figure S17, Supporting Information). The intracellular concentrations of Cu^2+^ and Mg^2+^ were quantified following incubation of the nanocomposites with the cells for varying time intervals. The results indicated a progressive increase in the ion content within the cells over time, thereby demonstrating the effective release of the materials into the cellular environment (Figure S18, Supporting Information). Bio‐TEM images were employed to investigate the potential of MgO_2_‐CuO_2_@HA NCs to induce cellular cuproptosis in 4T1 cells (Figure 3e). Following a 3‐h co‐incubation period, bio‐TEM images directly revealed the internalization of MgO_2_‐CuO_2_@HA NCs. In contrast to the intact architecture of the cells in the control group, the 4T1 cells treated with MgO_2_‐CuO_2_@HA NCs exhibited marked structural disruption accompanied by significant mitochondrial damage.

The production of intracellular ROS was evaluated by CLSM and flow cytometry using DCFH‐DA as a chemical probe (Figure 3f; Figure S19, Supporting Information). Compared to the other groups, the fluorescence intensity of MgO_2_‐CuO_2_@HA NCs treatment group was increased significantly, indicating its exceptional ROS generation capability. Actin filaments (F‐actin) were visualized using Actin Red staining to evaluate the morphological changes in 4T1 cells under different conditions. The F‐actin of cells in the control group exhibited a highly extended and well‐organized structure (Figure 3g). In contrast, F‐actin contracted and compacted, accompanied by evident cytoskeletal damage in the MgO_2_‐CuO_2_@HA NCs treatment group. Therefore, MgO_2_‐CuO_2_@HA NCs could cause injury to 4T1 cells. The integrity of the endosomal membrane was assessed using an acridine orange (AO) probe, which exhibits distinct green fluorescence in the cytoplasm and nucleus, while emitting red fluorescence when wrapped by intact lysosomes. After treatment with MgO_2_‐CuO_2_@HA NCs, the most significant reduction in red fluorescence was observed, indicating that the integrity of the endosomal membrane was severely damaged, which is consistent with oxidative stress‐induced membrane damage (Figure 3h). The mitochondrial function was detected by 5,5′,6,6′‐tetrachloro‐1,1′,3,3′‐tetraethylbenzimi‐dazolylcarbocyanine iodide (JC‐1) assay kit. Green fluorescence signifies depolarized and damaged mitochondrial membranes, whereas red fluorescence indicates polarized and healthy mitochondria. The 4T1 cells treated with MgO_2_‐CuO_2_@HA NCs showed the highest green/red fluorescence intensity ratio, suggesting significant mitochondrial damage (Figure 3i; Figure S20, Supporting Information). As a result, the mitochondrial damage caused by MgO_2_‐CuO_2_@HA NCs leads to an evident decrease of intracellular adenosine triphosphate (ATP) level (Figure S21, Supporting Information). The production of H_2_O_2_ was monitored using a fluorescent probe that generated green fluorescence upon reaction with H_2_O_2_, as illustrated in Figure 3j. The fluorescence intensity analysis revealed that the H_2_O_2_ production could be detected in all treatment groups, and compared to the other groups, the MgO_2_‐CuO_2_@HA NCs group exhibited the highest fluorescence intensity, indicating their superior H_2_O_2_ self‐supplying capability. In addition, the MgO_2_‐CuO_2_@HA NCs group presented superior GSH depletion compared to the other experimental groups, as evidenced by the GSH staining assay (Figure 3k). To further explore the tumor cell‐killing capability of MgO_2_‐CuO_2_@HA NCs, two complementary cell death assays were conducted. Calcein‐AM and propidium iodide (PI) double‐staining assays demonstrated the prominent anti‐tumor efficacy of MgO_2_‐CuO_2_@HA NCs (Figure 3l). In addition, the flow cytometric analysis using Annexin V‐FITC and PI co‐staining result indicated that the MgO_2_‐CuO_2_@HA NCs group had a significantly higher apoptotic ratio (59.30%) than that of the MgO_2_ nanosheet (41.03%) and CuO_2_ nanodot (31.86%) groups (Figure 3m). Furthermore, we investigated the effect of MgO_2_‐CuO_2_@HA NCs treatment on cell migration using scratch wound‐healing assays. As shown in Figure S22 (Supporting Information), the most significant inhibition of cell migration was detected after 12 and 24 h of MgO_2_‐CuO_2_@HA NCs treatment, whereas the control group demonstrated a negligible impact on cell migration.

**Figure 4:**
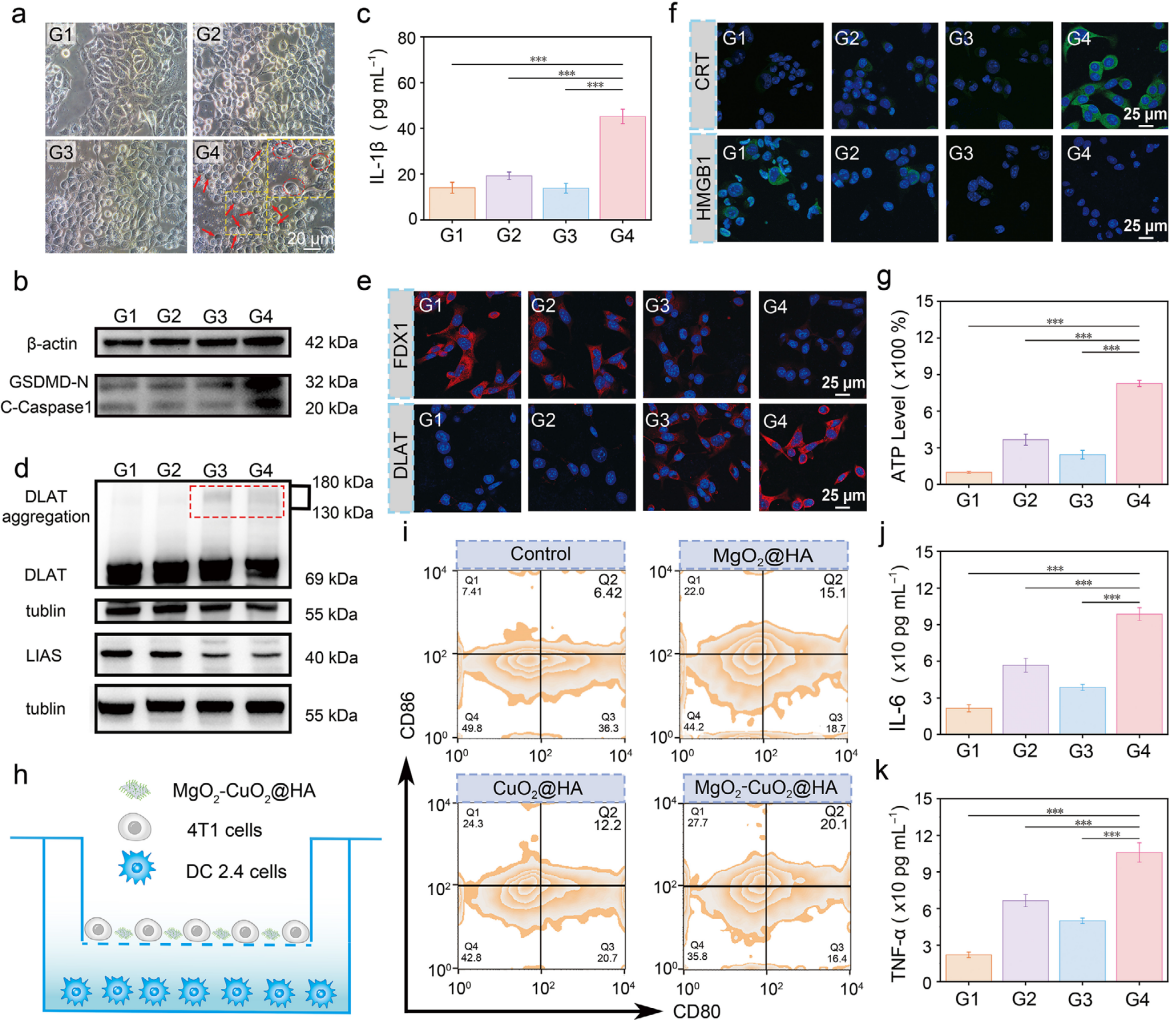
a) Representative bright field microscopy images of 4T1 cells after various treatments (the arrows indicate pyroptotic cells). b) The detection of pyroptosis‐associated proteins in 4T1 cells with various treatments using Western blot analysis, including GSDMD‐N and C‐Caspase‐1. c) ELISA measurement of IL‐1β in the extracellular supernatant of 4T1 cells with different treatments (*n *= 5, mean ± S.D.). d) Western blot evaluation of DLAT and LIAS expression after diverse treatments. e) CLSM images of FDX1 subcellular redistribution and DLAT exposure in 4T1 cells with diverse treatments. f) CLSM images of CRT and HMGB1 exposure on 4T1 cells after different treatments. g) ATP released in the extracellular supernatant of 4T1 cells after different treatments (*n *= 5, mean ± S.D.). h) Schematic illustration of the transwell co‐culture assay for evaluating DCs maturation induced by 4T1 cells with different treatments. i) The evaluation of DCs maturation was performed by analyzing CD80/CD86 expression via flow cytometry. ELISA measurement of j) IL‐6 and k) TNF‐α secreted in the extracellular supernatant of DC2.4 cells upon various treatments (*n *= 5, mean ± S.D.). The groups were divided into (G1) Control, (G2) MgO_2_@HA, (G3) CuO_2_@HA, and (G4) MgO_2_‐CuO_2_@HA NCs. Statistical significance was calculated using Student's *t‐*test: *p < 0.05, **p < 0.01, and ***p < 0.001.

Based on the remarkable tumor cell‐killing efficacy of MgO_2_‐CuO_2_@HA NCs, we conducted an in‐depth investigation of their potential cell death mechanism. Considering the significant production of ROS, we speculated that MgO_2_‐CuO_2_@HA NCs might induce pyroptosis, a type of programmed inflammatory cell death. To observe the changes in cell morphology, bright‐field images of 4T1 cells were captured after treatment with various formulations (**Figure** **4**a). In the MgO_2_‐CuO_2_@HA NCs group, many 4T1 cells exhibited prominent bubble‐like structures (marked with red arrows), whereas the 4T1 cells in the control group retained their normal structure. This observation suggests that MgO_2_‐CuO_2_@HA NCs could induce the pyroptosis‐associated morphological alterations in 4T1 cells. Western blotting (WB) analysis was performed to assess the protein expression levels during pyroptosis (Figure 4b). Excessive ROS can activate caspase‐1, which subsequently cleaves GSDMD into N‐GSDMD, inducing the formation of cellular membrane pores, thereby facilitating pyroptosis. As anticipated, the expression of GSDMD‐N and cleaved caspase‐1 (C‐Caspase‐1) in the MgO_2_‐CuO_2_@HA NCs treatment group was significantly higher than that in the other groups, suggesting that MgO_2_‐CuO_2_@HA NCs can activate the caspase‐1/GSDMD‐dependent pyroptosis pathway. Moreover, interleukin‐1β (IL‐1β), a typical pro‐inflammatory cytokine released during pyroptosis, exhibited the highest release after treatment with MgO_2_‐CuO_2_@HA NCs (Figure 4c). After the 4T1 cells were incubated with CuO_2_@HA nanodots and MgO_2_‐CuO_2_@HA NCs, significantly enhanced DLAT oligomer expression was observed in the larger protein molecular weight bands could be found, demonstrating that Cu^2+^ release induced DLAT aggregation. The expression of lipoic acid synthetase (LIAS) in the MgO_2_‐CuO_2_@HA NCs treatment group was significantly lower than that in the other groups (Figure 4d). Furthermore, the immunofluorescence staining images of Ferredoxin 1(FDX1) and DLAT also illustrated that MgO_2_‐CuO_2_@HA NCs could trigger the depletion of FDX1 and the aggregation of DLAT by releasing a considerable amount of Cu^2+^ (Figure 4e). These alterations indicate that MgO_2_‐CuO_2_@HA NCs can effectively induce carcinogenesis in tumor cells.

To validate the immunogenic effect of MgO_2_‐CuO_2_@HA NC‐induced cell death, we employed immunofluorescence assays to detect the characteristic ICD markers, specifically CRT and HMGB1. As shown in Figure 4f, compared to the other experimental groups, 4T1 cells treated with MgO_2_‐CuO_2_@HA NCs showed the highest surface exposure to CRT, and HMGB1 was almost completely transferred to the extracellular space. Furthermore, the extracellular ATP level in the MgO_2_‐CuO_2_@HA NCs treatment group was approximately eightfold higher than that in the control group (Figure 4g). These findings collectively provide compelling evidence that MgO_2_‐CuO_2_@HA NCs can induce substantial ICD effects through cuproptosis and pyroptosis‐mediated pathways. To evaluate the adaptive immune response induced by MgO_2_‐CuO_2_@HA NCs, the maturation of in vitro dendritic cells (DCs) was examined by establishing a transwell model using DC2.4 cells and 4T1 cells subjected to various treatments (Figure 4h). Maturation of these DCs was then assessed by measuring the percentages of CD80 and CD86 costimulatory molecules using flow cytometry. As shown in Figure 4i, the MgO_2_‐CuO_2_@HA NCs treatment group exhibited a significantly higher proportion of mature DCs (20.1%) than the other groups. Subsequently, the secretion profiles of immunostimulatory cytokines were evaluated by enzyme‐linked immunosorbent assay (ELISA), focusing on interleukin‐6 (IL‐6) and tumor necrosis factor‐α (TNF‐α) levels (Figure 4j,k). The expression of TNF‐α (4.8‐fold) and IL‐6 (4.6‐fold) in the MgO_2_‐CuO_2_@HA NCs group was significantly upregulated compared with the control group, demonstrating that ICD induced by MgO_2_‐CuO_2_@HA NCs can enhance the production of pro‐inflammatory cytokines. In addition, Mg^2+^ released from 4T1 cells after treatment with MgO_2_‐CuO_2_@HA NCs promoted the conformational activation of LFA‐1 on CD8^+^ T cells by binding to the metal‐ion‐dependent adhesion site, thereby facilitating the transition from a bent to an extended open‐headpiece conformation and enhancing T cell adhesion, signaling, and effector functions (Figure S23, Supporting Information). These data collectively verify that MgO_2_‐CuO_2_@HA NCs possess potent immunostimulatory properties and induce a comprehensive immunogenic response through multiple activation pathways.

**Figure 5:**
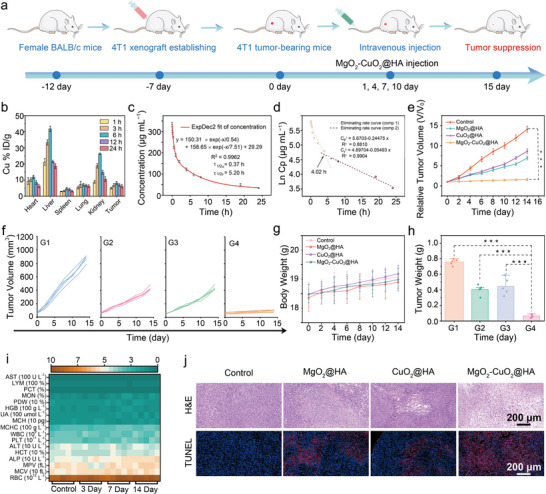
a) Schematic description of in vivo anticancer therapeutic experiments. b) The distribution of Cu^2^⁺ in main organs and tumors of representative mice at different time intervals after the intravenous administration of MgO_2_‐CuO_2_@HA NCs. c) The blood circulation profile and d) systemic clearance kinetics of MgO_2_‐CuO_2_@HA NCs with various injection time points (*n *= 5, mean ± S.D.). e) The relative tumor volume, f) tumor volume, and g) body weight of mice in each treatment group. h) The weight of excised tumors in different treatment groups after 14 days of treatment (*n* = 5, mean ± S.D.). i) Hematological parameters and biochemical indicators of mice after intravenous injection of PBS and MgO_2_‐CuO_2_@HA NCs over different time periods (*n* = 5). j) H&E and TUNEL staining assays of tumor tissues sections from various treatment groups. The experimental groups including (G1) Control, (G2) MgO_2_@HA, (G3) CuO_2_@HA, and (G4) MgO_2_‐CuO_2_@HA NCs. Statistical significance was calculated using Student′s *t‐*test: *p < 0.05, **p < 0.01, and ***p < 0.001.

The in vivo therapeutic efficacy of the MgO_2_‐CuO_2_@HA NCs was further evaluated using female BALB/c mice implanted with 4T1 tumors (**Figure** **5**a). Initially, the blood compatibility of MgO_2_‐CuO_2_@HA NCs was assessed to ensure their biosafety for potential therapeutic applications. The hemolysis test results indicated that the hemolytic effect of MgO_2_‐CuO_2_@HA NCs was almost negligible, indicating that the MgO_2_‐CuO_2_@HA NCs had good hemocompatibility (Figure S24, Supporting Information). As shown in Figure 5b, an elevated concentration of Cu^2+^ was detected in the heart, spleen, liver, lung, kidney, and tumor tissues, illustrating the in vivo distribution of MgO_2_‐CuO_2_@HA NCs. Notably, after 6 h of MgO_2_‐CuO_2_@HA NCs administration, the concentration of Cu^2+^ in tumor tissue reached 8.16% ID g^−1^, and remained at a relatively high level of 6.14% ID g^−1^ even after 24 h. Then, the behavioral characteristics of MgO_2_‐CuO_2_@HA NCs in the blood circulation were evaluated through pharmacokinetic analysis (Figure 5c). The circulation of MgO_2_‐CuO_2_@HA NCs in the bloodstream followed the traditional two‐compartment model, with the half‐lives determined to be *τ*_1/2α_ = 0.37 h and *τ*_1/2β_ = 5.2 h, respectively, which was conducive to achieving efficient tumor accumulation. The distribution rate constant of MgO_2_‐CuO_2_@HA NCs in the first phase was −0.24475 µg mL^−1^ h^−1^, which increased to an elimination rate constant of −0.05493 µg mL^−1^ h^−1^ after a 4.02‐h time interval (Figure 5d).

We further examined the tumor‐suppressive efficacy of MgO_2_‐CuO_2_@HA NCs in a 4T1 tumor‐bearing mouse model. Based on the relative tumor volume growth curves, the MgO_2_‐CuO_2_@HA NCs treatment group exhibited the most notable tumor growth inhibition, with a suppression rate of 88% (Figure 5e,f). As shown in Figure 5g, the mice in all treatment groups showed a stable weight gain trend during the treatment period, and no distinct organ damage was observed after the diverse treatments (Figures S25,S26, Supporting Information). The mean weight of the dissected tumor tissues, together with the analysis of associated digital images, further confirmed the apparent anti‐tumor treatment efficacy of MgO_2_‐CuO_2_@HA NCs (Figure 5h; Figure S27, Supporting Information). Moreover, the survival rate of mice in the MgO_2_‐CuO_2_@HA NCs group remained at ≈80% even after 45 days of treatment, which was significantly higher than that in the other groups (Figure S28, Supporting Information). To assess the biocompatibility of MgO_2_‐CuO_2_@HA NCs for possible practical applications, routine blood and biochemical tests were conducted on healthy BALB/c mice after intravenous injection of MgO_2_‐CuO_2_@HA NCs. During the evaluation period, all hematological parameters remained within normal ranges and exhibited no notable differences compared to the control group, suggesting that MgO_2_‐CuO_2_@HA NCs exerted a negligible effect on the hematological system. Furthermore, no significant abnormalities in kidney or liver function were observed before or after MgO_2_‐CuO_2_@HA NCs administration (Figure 5i). Moreover, hematoxylin and eosin (H&E) staining along with TdT‐mediated dUTP nick‐end labeling (TUNEL) staining images were performed to assess the apoptosis of tumor cells following different treatments. As shown in Figure 5j, the mice injected with MgO_2_‐CuO_2_@HA NCs exhibited the most significant level of tumor cell damage compared to the other groups. These findings indicate that MgO_2_‐CuO_2_@HA NCs possess satisfactory biosecurity for potential therapeutic applications.

**Figure 6:**
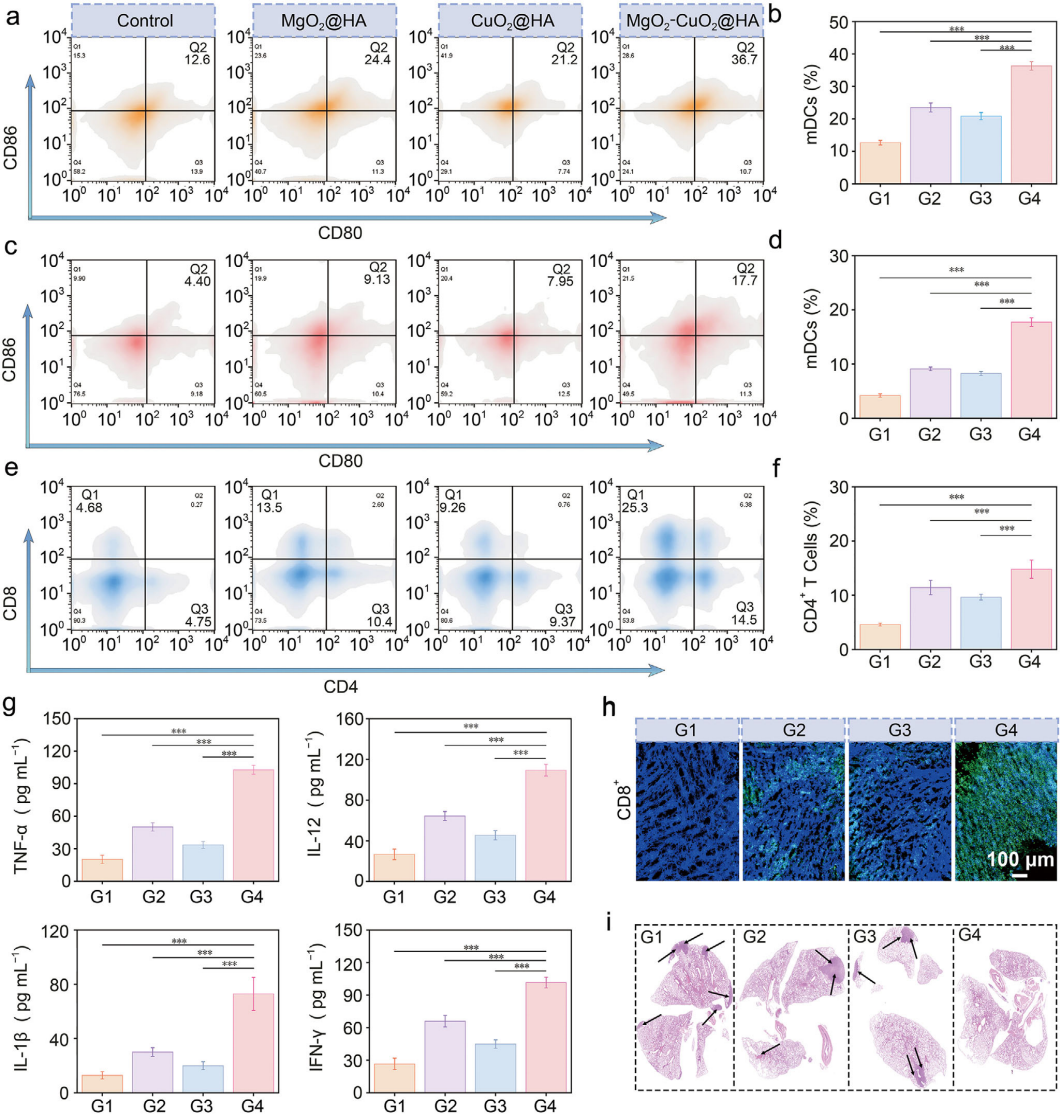
a) The flow cytometry data and b) quantitative analysis of matured DCs in spleens of representative mice after receiving different treatments (*n *= 5, mean ± S.D.). c) The evaluation of matured DCs in lymph nodes using flow cytometry. d) The corresponding quantitative analysis of matured DCs in lymph node (*n *= 5, mean ± S.D.). e) Flow cytometry data of CD4 and CD8 T cells in tumor tissues after 14 days of various treatments. f) The quantitative analysis of CD4^+^ T cells based on flow cytometry detection (*n *= 5, mean ± S.D.). g) The levels of cytokines TNF‐α, IL‐12, IL‐1β, and IFN‐γ in mice were evaluated by ELISA assay following various treatments (*n *= 5, mean ± S.D.). h) CD8^+^ staining images of tumor sections collected from representative mice in different treatment groups. i) H&E staining of representative lung tissue sections on anti‐metastatic effect. The groups were set as (G1) Control, (G2) MgO_2_@HA, (G3) CuO_2_@HA, and (G4) MgO_2_‐CuO_2_@HA NCs. Statistical significance was calculated using Student′s *t*‐test: *p < 0.05, **p < 0.01, and ***p < 0.001.

The induction of tumor ICD caused by cuproptosis and pyroptosis has been confirmed to stimulate anti‐tumor immune activity, thereby promoting cancer immunotherapy. To explore this mechanism, changes in critical indicators of the anti‐tumor immune response activated by MgO_2_‐CuO_2_@HA NCs were examined using flow cytometry. Consistent with the results of in vitro experiments, mice treated with MgO_2_‐CuO_2_@HA NCs exhibited the highest percentage of DCs maturation in the spleen compared with other groups (**Figure** **6**a,b). Mature DCs are essential for antigen presentation, which stimulates the growth of naïve T cells and triggers an adaptive immune response. Meanwhile, the MgO_2_‐CuO_2_@HA NCs treatment group also facilitated a greater number of DC maturation in the lymph nodes (Figure 6c,d). To further validate the immunotherapeutic effect of MgO_2_‐CuO_2_@HA NCs, the infiltration of T cells into tumor tissues was thoroughly assessed. As shown in Figure 6e,f, the proportions of CD4^+^ and CD8^+^ T cells in the MgO_2_‐CuO_2_@HA NCs treatment group were 14.8% and 24.5%, respectively, which were 3.2‐fold and 5.3‐fold higher than those in the control group (Figure S29, Supporting Information). Additionally, memory T cells (CD8^+^CD44^+^CD62L) were evaluated to confirm the significant immune memory effect after MgO_2_‐CuO_2_@HA NCs treatment (Figure S30, Supporting Information). The results showed that the proportion of memory T cells in the MgO_2_‐CuO_2_@HA group reached 21.32%, representing a 16.4‐fold increase compared with the control group. Furthermore, the levels of immune‐related cytokines IL‐6, IL‐12, TNF‐α, and interferon‐γ (IFN‐γ) in the serum were significantly elevated in the MgO_2_‐CuO_2_@HA NCs group, suggesting a robust stimulation of T cell‐mediated immune response (Figure 6g; Figure S31, Supporting Information). Moreover, the upregulation of IL‐1β further explained the occurrence of pyroptosis. In addition, after treatment with MgO_2_‐CuO_2_@HA NCs, the infiltration of CD8^+^ T cells at the tumor sites was distinctly higher than that in the other groups, as shown in Figure 6h. Ultimately, owing to the potent anti‐tumor immune response mediated by MgO_2_‐CuO_2_@HA NCs, the tumor cells scarcely metastasized to the lung tissue (Figure 6i).

## 3 Conclusion

In summary, a novel class of bimetallic peroxide nanocomposites with a uniform morphology and excellent dispersibility was successfully developed via a straightforward method. These MgO_2_‐CuO_2_@HA NCs exhibited targeted delivery capacity to tumor cells owing to modification with HA and the release of significant amounts of Mg^2+^, Cu^2+^, and H_2_O_2_ upon endocytosis. The release of Cu^2+^ catalyzes the self‐generated H_2_O_2_ to produce substantial •OH through a Fenton‐like reaction. Cu^2+^ overloading results in the aggregation of DLAT and LIAS reduction, thereby inducing cuproptosis in tumor cells. Additionally, increased ROS production activates pyroptosis through the caspase‐1/GSDMD pathway. As a result, MgO_2_‐CuO_2_@HA NCs could not only triggered cytoproptosis and pyroptosis, but also initiated ICD in tumor cells, thereby sensitizing the anti‐tumor adaptive immune response. The released Mg^2+^ promotes the conformational activation of LFA‐1, thereby enhancing the activation of CD8^+^ T cells. Furthermore, MgO_2_‐CuO_2_@HA NCs demonstrated long‐term anti‐tumor immune therapeutic effects and effectively inhibited the metastasis of tumor cells. Overall, MgO_2_‐CuO_2_@HA NCs effectively induced cuproptosis and pyroptosis in tumor cells and activated anti‐tumor adaptive immune responses, offering novel insights and valuable references for the application of metal peroxides in tumor immunotherapy.

## 4 Experimental Section

### Synthesis of MgO_2_ Nanosheets

MgO_2_ nanosheets were synthesized via a microemulsion approach. First, 10 mmol of MgCl_2_·6H_2_O was dissolved into 5 mL of deionized water under ultrasonication to form a homogeneous solution. Next, 60 mL of cyclohexane was added to the mixture under magnetic stirring at 500 rpm, followed by the dropwise addition of 2 mL of CO‐520 surfactant to establish a reverse micelle microemulsion system. After stirring for 30 min, 5 mL of NaOH aqueous solution (0.25 m) was slowly added into the above solution and stirred continuously for 20 min. Subsequently, 5 mL of H_2_O_2_ (30%) was added to the mixture, followed by continuous stirring at room temperature for 30 min. At last, 220 mL of anhydrous ethanol was introduced to destabilize the reverse microemulsion system. The product was collected by centrifugation, washed with anhydrous ethanol for three times to remove residual surfactants, and freeze‐dried under vacuum to obtain the final MgO_2_ nanosheets.

### Synthesis of CuO_2_ Nanodots

First, 0.2 mmol of Cu(CH_3_COO)_2_·H_2_O was dispersived into 20 mL aqueous solution containing 100 mg of PEI. The mixture was dispersed uniformly via ultrasonication and stirred vigorously at room temperature for 0.5 h. Subsequently, 5 mL of aqueous H_2_O_2_ (30%) was added dropwise, and the reaction was allowed to proceed under stirring at room temperature for 24 h. The resulting product was isolated by centrifugation, washed 2–3 times with deionized water to remove unreacted precursors, and freeze‐dried under vacuum to obtain the CuO_2_ nanodots.

### Synthesis of MgO_2_‐CuO_2_@HA Nanocomposites (NCs)

The prepared MgO_2_ nanosheets and CuO_2_ nanodots were dispersed into 50 mL of anhydrous alcohol at a mass ratio of 1:3, and 1 mg mL^−1^ of NaHA was added and stirred overnight. The resulting products were collected by centrifugation and rinsed three times with anhydrous ethanol, the products were freeze‐dried. To isolate the impact of sodium hyaluronate modification on the properties of individual nanomaterials in both in vitro cellular and in vivo experiments. The MgO_2_@HA nanosheets and CuO_2_@HA nanodots were separately prepared using an analogous protocol.

### Cell Line and Animals

Mouse NCTC clone 929 cell line (L cell, L929, RRID: CVCL_0462), mouse breast cancer cell line (4T1, RRID: CVCL_0125), and mouse dendritic cell line (DC2.4, RRID: CVCL_J409) were purchased from Thermo Fisher in January 2025. All three cell lines were free from contamination. All in vivo experiments approved by the Ethics Committee of the Affiliated Tumor Hospital of Nantong University (2024‐001).

### Cytotoxicity Analysis

To evaluate the biocompatibility and cytotoxicity of MgO_2_‐CuO_2_@HA NCs were examined using a standard thiazole tetrazolium (MTT) assay in L929 cells and 4T1 cell lines. 4T1 cells were seeded in a 96‐well plate and incubated overnight, subsequently treated with Control, MgO_2_@HA, CuO_2_@HA, and MgO_2_‐CuO_2_@HA NCs with various concentrations (25, 50, 100, 200, 250, and 300 µg mL^−1^). After 24 h of incubation, PBS was used to wash the cells, then 20 µL of MTT solution (5 mg mL^−1^) was added into each well. Following a 4 h incubation period, the formazan crystals were solubilized by adding 150 µL of dimethyl sulfoxide (DMSO) per well, followed by gentle shaking for 10 min. The absorbance at 490 nm was detected for calculating the cell viability. Similarly, L929 cells were treated with MgO_2_@HA, CuO_2_@HA, and MgO_2_‐CuO_2_@HA NCs with different concentrations (25, 50, 100, 200, 250, and 300 µg mL^−1^) for 24 h, and other experimental procedures were consistent with the cytotoxicity test.

### Mitochondrial Integrity Assay

4T1 cells were cultured in 6‐well plates and allowed to adhere for 24 h. Following this, the cells received different treatments, containing Control, MgO_2_@HA, CuO_2_@HA, and MgO_2_‐CuO_2_@HA NCs. The cells were further incubated for 4 h and subsequently stained with JC‐1 dye for 20 min according to the protocol provided. The cells were then cleaned with PBS to eliminate excess dye and then subjected to Hoechst 33 342 for 15 min. CLSM was used to observe the status of mitochondrial. In parallel, flow cytometry analysis was conducted on the collected cells post‐treatment, suspended cells underwent a second incubation with JC‐1 dye for another 20 min. Ultimately, quantitative assessment was carried out through flow cytometry.

### Living/Dead Cells Staining

4T1 cells were inoculated in 6‐well plates and incubated for 24 h. Then cells were treated with different formulations, including Control, MgO_2_@HA, CuO_2_@HA, and MgO_2_‐CuO_2_@HA NCs, respectively. After incubating with the material for 4 h, the excess material was cleaned with PBS, and the mixture of PI (500 µL, 4 µm) and Calcin‐AM (300 µL, 3 µm) was added and incubated for 30 min. The cells were achieved, and CLSM was employed to analyze the fluorescence intensity.

### Assessment of Lysosomal Membrane Integrity

4T1 cells were inoculated randomly in a 6‐well plate at a density of 1 × 10^5^ cells per well and allowed to adhere for 24 h. The cells were washed twice with PBS and then treated with the following conditions: control, MgO_2_@HA, CuO_2_@HA, and MgO_2_‐CuO_2_@HA NCs, respectively. After 4 h of incubation, 1 mL of acridine orange (AO) with a concentration of 10 µm was added and co‐incubated for 20 min. The cells were rinsed with PBS three times, collected, and visualized by CLSM.

### In Vivo Antitumor Effect

A 4T1 tumor‐bearing mouse model was established by subcutaneous injection of 4T1 cancer cells. (100 µL containing 5 × 10^6 ^cells) into the right posterior flank of each mouse. Subsequent experiments commenced when the tumor size grew to ≈80 mm^3^, the mice were randomly divided into four groups (*n* = 5), including Control, MgO_2_@HA, CuO_2_@HA, and MgO_2_‐CuO_2_@HA NCs, respectively. Mice in each group received the intravenous injection of various formulations at a dose of 10 mg kg^−1^ (100 µL). The body weight and tumor dimensions of each mouse were recorded every two days during the treatment period. Tumor volume was calculated using the formula:

(1) $$\mathrm{Tumor}\;\mathrm{volume}=\frac{W^{2}L}{2}$$

where the *L* (mm) and *W* (mm) denote the width and length of the tumor. At 14 days post‐treatment, mice were euthanized, and tumors were excised for photographic documentation and weighing to assess therapeutic efficacy.

### Histological Examination

Following 14 days of treatment, histological analysis was carried out. The mice in each treatment group were sacrificed, and the main organs and tumors were harvested for hematoxylin and eosin (H&E) staining assay. The stained tissue slices were imaged by utilizing the CLSM.

### TUNEL Assay

The APO‐BrdU TUNEL assay kit was used to identify apoptotic cells following the manufacturer's protocol. First, tissue sections were fixed in PBS supplemented with 4% paraformaldehyde at room temperature for a duration of 10 min. After rinsing with PBS, the sections were treated with a permeabilization solution for 2 min. Then, the slices were washed again and incubated with the TUNEL reaction mixture under humid conditions at 37 °C in darkness for 60 min. Following a gentle rinse with PBS, the sections were mounted and prepared for further analysis via CLSM.

### Statistical Analysis

Quantitative data were presented as mean ± S.D. The Student's *t*‐test was employed for mean comparison, statistical significance was assumed at a value of *p < 0.05, **p < 0.01, and ***p < 0.001.

## Conflict of Interest

The authors declare no conflict of interest.

## Supporting information



Supporting Information

## Data Availability

The data that support the findings of this study are available in the supplementary material of this article.
