# Supplementary material for: Bimetallic Peroxide Nanocomposites‐Driven Redox Dyshomeostasis to Activate Sequential Cuproptosis and Pyroptosis for Amplified Tumor Immunotherapy
Source: Adv Sci (Weinh). 2025 Nov 23;13(18):e12470. doi: 10.1002/advs.202512470 (PMC13042952; doi:10.1002/advs.202512470)
Supplement: Supplementary file 1 — Supporting Information [file ADVS-13-e12470-s001.docx]

Supporting Information

**Bimetallic Peroxide Nanocomposites-Driven Redox Dyshomeostasis to Activate Sequential Cuproptosis and Pyroptosis for Amplified Tumor Immunotherapy**

*Guanting He, Haixia Zhu, Peipei Kang, Lili Feng*, Chenghao Yu, Yanlin Zhu, Bin Liu*, Zhengxiao Guo*, Piaoping Yang**

**Experimental Section**

***Chemicals and Reagents*.** All of the chemicals were used without further purification. Sodium hydroxide (NaOH) and magnesium chloride hexahydrate (MgCl_2_·6H_2_O) were obtained from Aladdin Reagent. Hydrogen peroxide (H_2_O_2_, 30%) was obtained from Beijing Chemical Works (Beijing, China). Polyoxyethylene (5) nonylphenylether, branched (CO-520) was obtained from Sigma-Aldrich Co., Ltd. Cupric acetate monohydrate (Cu(CH_3_COO)_2_·H_2_O) was obtained from Xilong Chemical Reagent Co, Ltd. Other chemicals including glutathione (GSH), 5,5′-dithiobis (2-nitrobenzoic acid) (DTNB), titanic sulfate (Ti(SO_4_)_2_), sodium hyaluronate (NaHA), and o-phenylenediamine (OPD), 3,3′,5,5′-tetramethylbenzidine (TMB), Hoechst 33342, 3-[4,5-dimethylthiazol-2-yl]-2,5-diphenyltetrazolium bromide (MTT), methylene blue (MB), Calcein-AM, propidium iodide (PI), and 2′,7′-dichlorofluorescin diacetate (DCFH-DA) were obtained from Sigma-Aldrich. Fetal bovine serum (FBS), RPMI 1640, JC-1 staining probe, 0.25% trypsin-EDTA, and the Annexin-V FITC/PI assay kit were achieved from Beyotime Institute of Biotechnology in Haimen, China. Additionally, the glutathione assay kit was sourced from Wanleibio Co., Ltd. Dihydrolipoamide S–acetyltransferase (DLAT) polyclonal antibody and lipoic acid synthetase (LIAS) polyclonal antibody were obtained from Proteintech (Wuhan, China). Cleaved caspase-1 (Absin, abs143596) and gasdermin D (GSDMD, Rabbit Polyclonal GSDMDC1 Antibody, Novus, NBP2-33422). PE anti-mouse CD4 (cat. 100408), APC anti-mouse CD8a (cat. 100712), FITC anti-mouse CD3ε (cat. 100305), FITC anti-mouse CD11c (cat. 117305), APC anti-mouse CD86 (cat. 105012), PE anti-mouse CD80 (cat. 104707), PE anti-mouse CD44 (cat. 103007), FITC anti-mouse CD62L (cat. 104405), and the enhanced ATP assay kit was sourced from Beyotime Biotechnology in Shanghai, China. TNF-α (BioLegend, cat. 1217202), IL-1β (Tianjin Anoric Biotechnology), IL-12p70 (BioLegend, cat. 1211202) and IL-6 (BioLegend, cat. 1210602) and IFN-γ (BioLegend, cat. 1210002) enzyme-linked immunosorbent assay (ELISA) kits were purchase from DAKEWE. Analytical grade was the standard for all other reagents, which were used as received.

***Characterizations*.** The transmission electron microscopy (TEM) image was captured using an FEI Tecnai T20 transmission electron microscope. The X-ray diffraction (XRD) pattern of the sample was analyzed using a Rigaku D/max-TTR-III diffractometer with Cu-Ka radiation (λ = 0.15405 nm) at 40 kV and 40 mA. Elemental quantification for specified components was carried out by inductively coupled plasma mass spectrometry (ICP-MS). X-ray photoelectron spectroscopy (XPS) measurements were carried out utilizing a Thermo Scientific ESCALAB 250XI instrument. The Zeta potential of the samples was assessed using a Malvern Zetasizer Nano ZS90. UV-1601 spectrophotometer was utilized to evaluate the UV–vis absorption spectrum of different samples. Electron spin resonance (ESR) spectra were recorded on a Bruker EMX1598 spectrometer. Atomic force microscopy (AFM) images were measured using a Veeco DI Nanoscope Multi-Mode V system. Fourier-transform infrared spectroscopy (FT-IR) analysis was conducted with a Bruker Tensor II instrument. Flow cytometry assays were performed employing a BD Accuri C6 flow cytometer, and fluorescence image was performed using a Leica TCS SP8 confocal laser scanning microscope.

***H_2_O_2_ released from*** ***MgO_2-_CuO_2_@HA NCs*.** Ti(SO_4_)_2_ served as an indicator for H_2_O_2_ detection, which transitions from colorless to yellow upon specific reaction with H_2_O_2_. 1 mg of MgO_2_-CuO_2_@HA NCs was mixed into 1 mL of Ti(SO_4_)_2_ aqueous solution with different pH values including 7.4, 6.5, and 5.5. The mixture was stirred for specified time periods (0, 5, 10, 15, and 30 min). After centrifugation, the supernatants were collected and blended with 1 mL of Ti(SO_4_)_2_ solution (1 mg mL^−1^). Subsequently, the absorbance of each mixture was measured spectrophotometrically at 410 nm. Furthermore, the UV–vis absorption spectra of the Ti(SO_4_)_2_ solution were evaluated after 30 min of treatment with various groups at pH = 5.5, including the MgO_2_, CuO_2_, and MgO_2_-CuO_2_@HA, respectively.

***Measurement of GSH depletion*.** The assessment of GSH consumption was conducted by quantifying the decrease in absorbance of glutathionylated DTNB at the wavelength of 412 nm. Briefly, MgO_2_-CuO_2_@HA NCs (500 μg mL^−1^) were incubated with 10 mM GSH. At various reaction time intervals, 1 mL of mixture solution was sampled and combined with DTNB (100 μL, 6 mM), the absorption spectrum of the resulting solution was analyzed using a UV–vis spectrophotometer. Also, MgO_2_-CuO_2_@HA NCs solutions with various concentrations were also performed to evaluate the consumption capability of GSH.

***Methylene blue (MB) degradation assay*.** To evaluate the catalytic activity of MgO_2_-CuO_2_@HA NCs, MB degradation assay was performed. Initially, 100 μL of MgO_2_-CuO_2_@HA NCs in water was mixed with 1 mL of MB solution (10 μg mL^−1^), resulting in a final solution concentration of 200 μg mL^−1^. H_2_O_2_ was added and adjusted to the concentration of 50 μM. The MgO_2_-CuO_2_@HA NCs were separated through centrifugation, and the absorbance spectrum of the supernatant was measured at various time intervals (0, 5, 10, 15, and 30 min) using a UV–vis spectrophotometer.

***Detection of •OH production*.** 1 mg of MgO_2_-CuO_2_@HA NCs were uniformly dissolved into 1 mL of pH-adjusted solutions (7.4, 6.5, and 5.5), and incubated under continuous stirring for varying durations (0.5, 1.5, 3, 6, and 12 h). For the TMB test, supernatants isolated *via* centrifugation were blended with 1 mL TMB aqueous solution, followed by the absorbance was measured using UV–vis spectrophotometer. Similarly, for the OPD assay, 1 mg of MgO_2_-CuO_2_@HA NCs was evenly dispersed into 1 mL of solutions with adjusted pH levels (7.4, 6.5, and 5.5) and incubated with constant stirring for different time periods (0.5, 1.5, 3, 6, and 12 h), The clarified supernatants were mixed with 1 mL of OPD solution, and analyzed for absorbance.

***ESR measurement*.** The detection of •OH was performed by using DMPO as a spin-trapping agent. The mixture of MgO_2_-CuO_2_@HA NCs (100 μL, 1 mg mL^−1^), DMPO (10 μL), and phosphate-buffered saline (340 μL, pH = 5.5) was prepared. Followed by H_2_O_2_ (100 mM, 50 μL) was added rapidly, an electron paramagnetic resonance spectroscopy was utilized to analyze the •OH generation capability.

***In vitro cellular uptake*.** The 4T1 cells were placed in 6-well plates and cultured for 24 h. FITC-labeled MgO_2_-CuO_2_@HA NCs solution (200 μg mL^–1^) was added and incubated for varying duration times (1, 2, and 4 h). Then, the cells were rinsed with PBS and then stained with Lyso-Tracker Red for 20 min and subsequently stained with Hoechst 33342 for another 15 min. Afterwards, the cells were washed with PBS, to remove the excess dyes and observed using CLSM.

***Intracellular ROS detection assay*.** After being cultured in 6-well plates for 24 h, 4T1 cells were rinsed with PBS and subjected to various treatments, including (G1) Control, (G2) MgO_2_@HA, (G3) CuO_2_@HA, and (G4) MgO_2_-CuO_2_@HA NCs. The final concentration of different samples was 200 μg mL^−1^. After 4 h of incubation, the cells were cleaned with PBS and further stained with DCFH-DA for 20 min under dark conditions. Finally, the cells was collected and analyzed by CLSM. For the detection of ROS by flow cytometry, the treated cells were trypsinized, centrifuged, and resuspended in PBS. Fluorescence intensity of the DCF was quantified using a flow cytometer.

***Apoptosis assay*.** To quantify apoptosis and necrosis induced by the materials, 4T1 cells were analyzed using an Annexin V-FITC/PI double-staining assay followed by flow cytometry. After incubation with different samples, 4T1 cells were stained with 5 μL PI and 95 μL Annexin V-FITC for 15 min. Finally, the stained cells in each group were assessed using flow cytometry.

***Cytoskeleton staining*.** 4T1 cells were plated in 6-well plate with the density of 1× 10^5^ cells per well and cultured for 24 h. The cells were treated with Control, MgO_2_@HA, CuO_2_@HA, and MgO_2_-CuO_2_@HA NCs, respectively. After 4 h of incubation, the nucleus was fixed with 4 % paraformaldehyde for 10 min. After washing, 1 % Triton X-100 was used to permeabilize them for 5 min, the nucleus was stained with Hoechst 33342 for 15 min, while F-actin filaments were labeled using Actin Red^TM^ for 20 min. Subsequently, the cells were collected, and their morphological features were analyzed using the CLSM.

***Cellular H_2_O_2_ detection*.** 4T1 cells were seeded in a 6-well plate and incubated for 24 h. Then, the cells were subjected to the following treatment conditions, Control, MgO_2_@HA, CuO_2_@HA, and MgO_2_-CuO_2_@HA NCs, respectively. To assess the intracellular H_2_O_2_ levels, the cells were incubated with the ROSGreen^TM^ H_2_O_2_ probe for 20 min in the dark. Following this, the cells were cleaned with PBS to remove any residual dye. The nuclei were labeled with Hoechst 33342 for 15 min. Lastly, the fluorescence intensity was analyzed using CLSM.

***Cellular GSH detection*.** 4T1 cells were inoculated in 6-well plates and incubated for 24 h. After attachment, the culture medium was discarded, and the wells were rinsed twice with PBS. The cells were subjected to the following treatments, including Control, MgO_2_@HA, CuO_2_@HA, and MgO_2_-CuO_2_@HA NCs, respectively. Intracellular GSH concentrations were detected using the commercial Thiol Tracker^TM^ Violet probe following the manufacturer's instructions. Finally, fluorescence images were captured using a CLSM.

***Intracellular ATP levels detection*.** A 6-well plate was used to seed 4T1 cells, which were then incubated for a day. The following day, the cells were treated with different ways, including Control, MgO_2_@HA, CuO_2_@HA, and MgO_2_-CuO_2_@HA NCs, respectively. After 4 h of incubation, the culture medium was removed, and residual nanomaterials were washed away. The cells were collected and ATP levels in each group were measured using ATP detection kit.

***Bio-transmission electron microscopy (bio-TEM)*.** 4T1 cells were seeded in culture plates and treated with culture medium and MgO_2_-CuO_2_@HA NCs (200 μg mL^−1^) for 4 h. Afterwards, the cells were rinsed multiple times with PBS and processed for bio-TEM analysis.

***Western blot*.** To detect DLAT, LIAS, GSDMD-N, and C-Caspase-1 *via* western blots, 4T1 cells were cultured and treated with various groups. The cells were then rinsed with PBS and harvested for western blot electrophoresis. Cells were lysed with RIPA buffer, the protein concentration was measured, the proteins were loaded onto the gel for electrophoresis, and then transferred onto polyvinylidene fluoride membranes for western blot analysis. Different specific antibodies were employed according to the requirements of the experiment. The protein bands were eventually detected by a chemiluminescence detection system.

***Calreticulin (CRT) and high-mobility group box 1 (HMGB1) expression detection*.** 4T1 cells were grown in 6-well culture plates and exposed to different treatments, containing Control, MgO_2_@HA, CuO_2_@HA, and MgO_2_-CuO_2_@HA NCs. After 4 h of incubation period, the supernatant from each well was collected and centrifuged for further analysis using an ELISA kit. Subsequently, the cells in the plates were rinsed with PBS, rapidly fixed, and and sealed overnight with the blocking solution. Afterwards, the cells were treated with anti-HMGB1 antibody for 60 min, washed three times with TBST solution and stained with secondary antibody. After secondary antibody staining, the cells were labeled with Hoechst 33342 and observed under a fluorescence microscope. In a similar manner, the cells were stained with anti-CRT antibody, and images were captured for further observation.

***In vitro dendritic cells (DCs) stimulation detection*.** To detect the DCs stimulation, DC2.4 cells was co-incubated with 4T1 cells in a transwell system. Following 4T1 cells were treated with various formulations and incubating for 12 h DCs were labeled with anti-CD11c, anti-CD86 and anti-CD80 antibodies, and analyzed by flow cytometry. Additionally, the suspension was gathered to measure TNF-α and IL-6 levels secreted by ELISA assay.

***Tumor mouse model*.** Female BALB/c mice (aged 4 weeks) were obtained from Beijing Vital River Laboratory Animal Co, Ltd. (Beijing, China) with the approval number 1100111084356. The animal experiments were conducted with the approval of ethics by the Ethics Committee of Harbin Medical University Affiliated Second Hospital (No.YJSDW 2024-267).

***Hemolysis test*.** 5 mL of blood sample was collected from mice *via* retro-orbital bleeding and anticoagulated with EDTA. The blood was then washed with PBS until the supernatant became clear. The purified red blood cells were resuspended in 10 mL of PBS. For testing, 1 mL of the diluted red blood cells was incubated with 100 μL of MgO₂-CuO₂@HA NCs at various concentrations for 3 h. And deionized water and PBS served as the positive control and negative control, respectively. The samples were then centrifuged at 1500 rpm for 3 min. Afterwards, the absorbance of the supernatant was determined with a UV–vis spectrophotometer. The hemolysis percentage was calculated as:

***In vivo blood circulation and biodistribution*.** To evaluate the pharmacokinetics and biodistribution of MgO_2_-CuO_2_@HA NCs, the tumor-bearing mice was intravenously injected with MgO_2_-CuO_2_@HA NCs at a dosage of 10 mg kg^−1^. 50 μL of blood samples were collected *via* tail vein at various post-injection time points (0.08, 0.17, 0.3, 0.5, 1, 2, 4, 6, 8, 19, and 24 h) for further analysis. The blood circulation half-life (τ_1/2_) of MgO_2_-CuO_2_@HA NCs was measured using ICP-MS and pharmacokinetic parameters were analyzed by two-compartment model. To investigate the biodistribution of MgO_2_-CuO_2_@HA NCs, which was administered into tumor-bearing mice at the aforementioned dosage level at 1, 3, 6, 12, and 24 h post-injection, mice were euthanized, and major organs (heart, liver, spleen, kidney, and lung) and tumors were harvested. Tissues were weighed, and dissolved in aqua regia and analyzed by ICP-MS to determine the concentration of Cu ions.

***In vivo antitumor immune effect*.** To evaluate the therapeutic efficacy of MgO_2_-CuO_2_@HA NCs in modulating antitumor immunity, the mice (n = 5 per group) were sacrificed after 14 days of various treatment. The tissues including lymph nodes, spleens, primary and abscopal tumors of the representative mice were collected, grinded in PBS, and filtered to gain single-cell suspension for further analysis. To assess DC maturation, spleen and lymph node cell suspensions were stained with FITC anti-mouse CD11c, APC anti-mouse CD86, and PE anti-mouse CD80 antibodies, and then analyzed using flow cytometry to evaluate DC maturation status. For the tumor-infiltrating T cells analysis, the cell suspensions from dissociated tumor tissues were stained with APC anti-mouse CD8a, PE anti-mouse CD4 and FITC anti-mouse CD3ε, and then measured by flow cytometry analysis identified T cells within the tumor microenvironment. For the memory T cells detection, the tumor cells were collected and stained by APC anti-CD8a, FITC anti-CD62L and PE anti-CD44. Flow cytometry was used to quantify memory T cell populations. Moreover, the isolate serums were gathered and quantified the levels of pro-inflammatory cytokines (IL-12, IL-6, TNF-α, IFN-γ, and IL-1β) using ELISA kits.


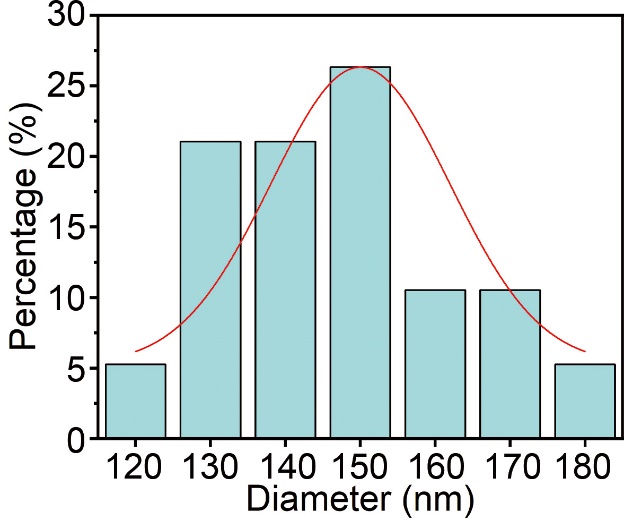


**Figure S1.** The size distribution of MgO_2_-CuO_2_@HA NCs.


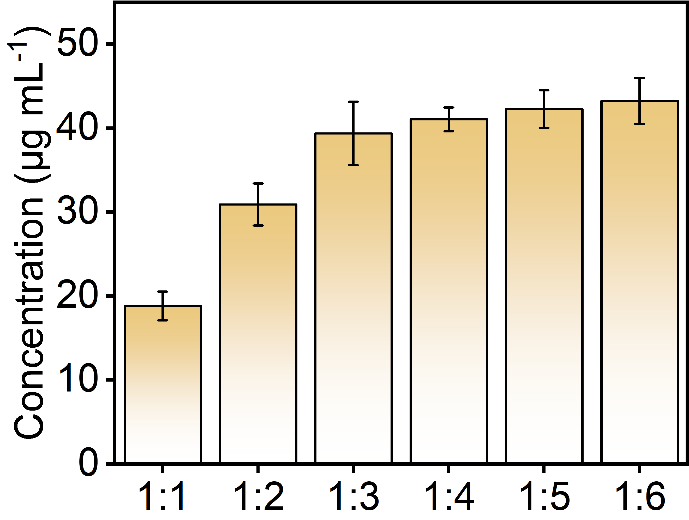


**Figure S2.** The release of Cu^2+^ from MgO_2_-CuO_2_ NCs composed of MgO_2_ nanosheets and CuO_2_ nanodots in different proportions after acidic treatment (pH = 5.5). Data are expressed as mean ± S.D. (n = 5).

**
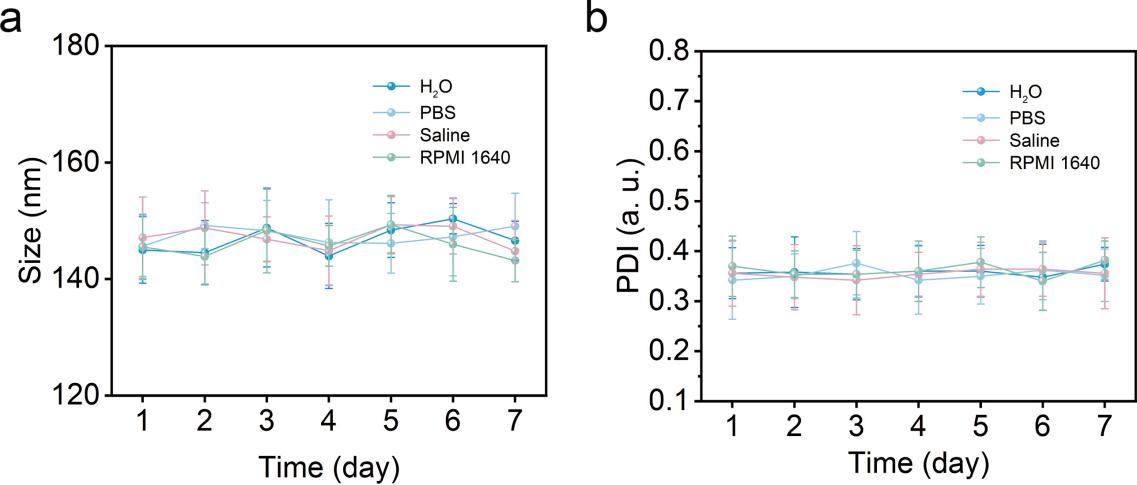
**

**Figure S3.** (a) Hydrodynamic dimension changes and (b) the PDI changes of MgO_2_-CuO_2_@HA NCs dispersed in different physiological solutions during 7 days of incubation (average PDI = 0.36). Data are expressed as mean ± S.D. (n = 5).

**
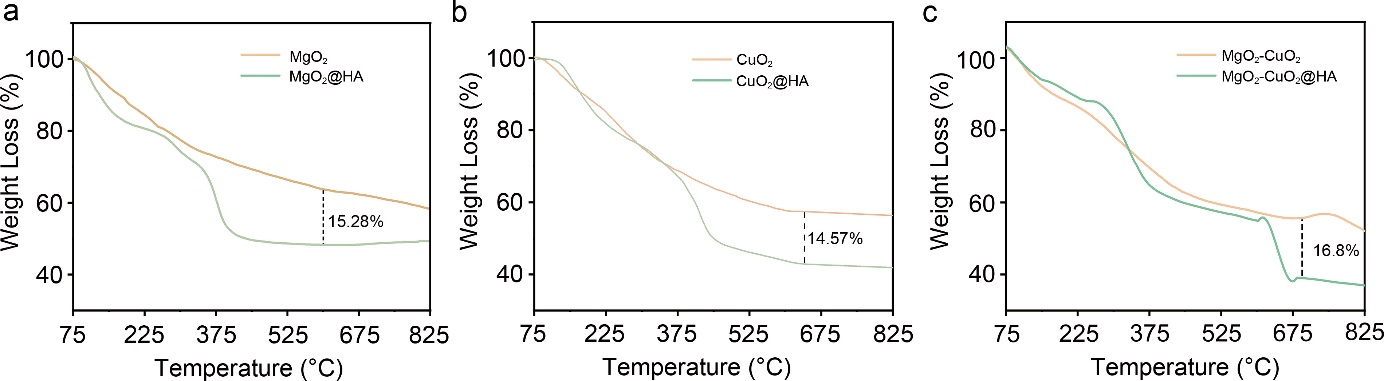
**

**Figure S4**. (a) TGA curves of (a) MgO_2_ and MgO_2_@HA, (b) CuO_2_ and CuO_2_@HA, and (c) MgO_2_-CuO_2_ and MgO_2_-CuO_2_@HA in the temperature range from 75 ℃ to 825 ℃.


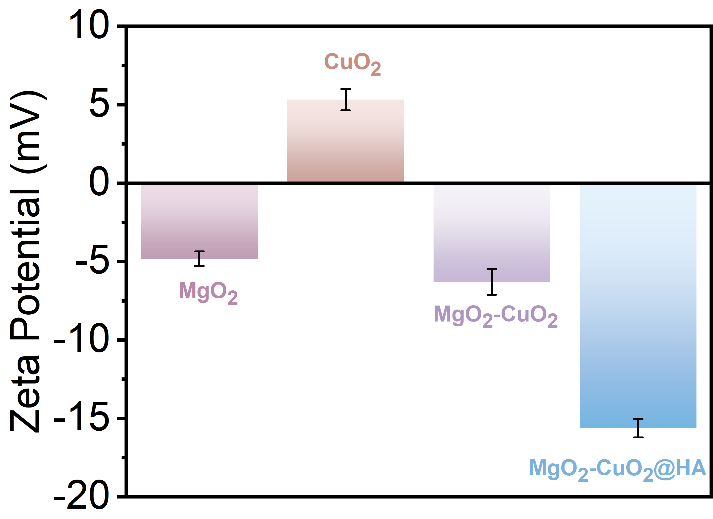


**Figure S5.** The Zeta potentials of different samples during the synthesis process. Data are expressed as mean ± S.D. (n = 5).


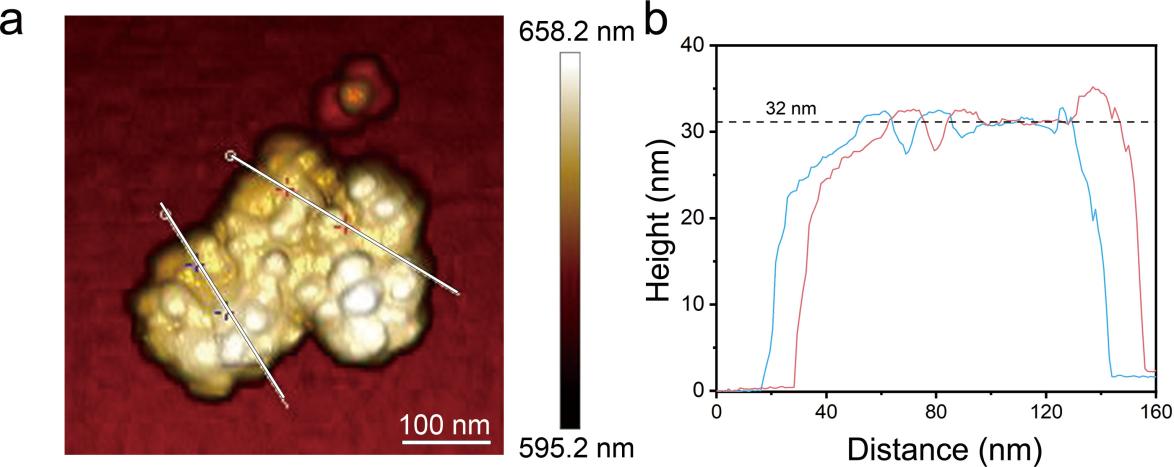


**Figure S6.** (a) AFM images of MgO_2_ nanosheets and (b) the thickness distribution of MgO_2_ nanosheets as measured by AFM image.


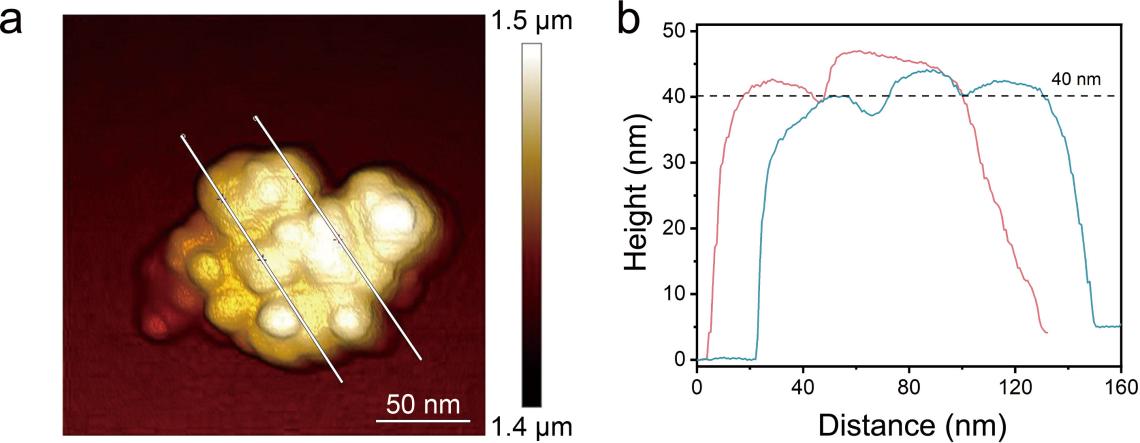


**Figure S7**. (a) AFM images and (b) the thickness distribution profile of MgO_2_-CuO_2_@HA NCs.


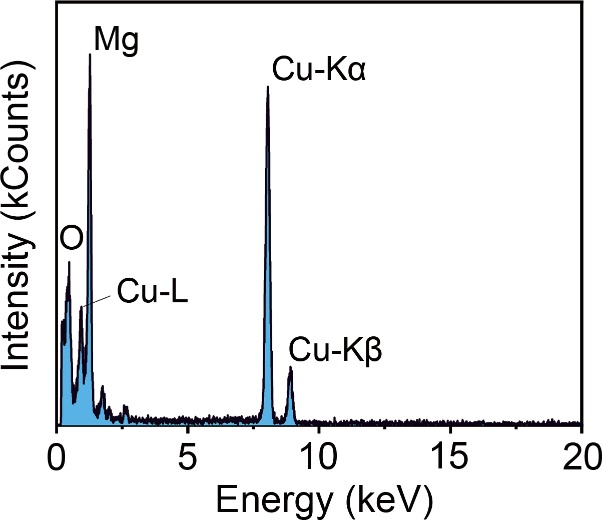


**Figure S8.** EDS survey spectrum of MgO_2_-CuO_2_@HA NCs.


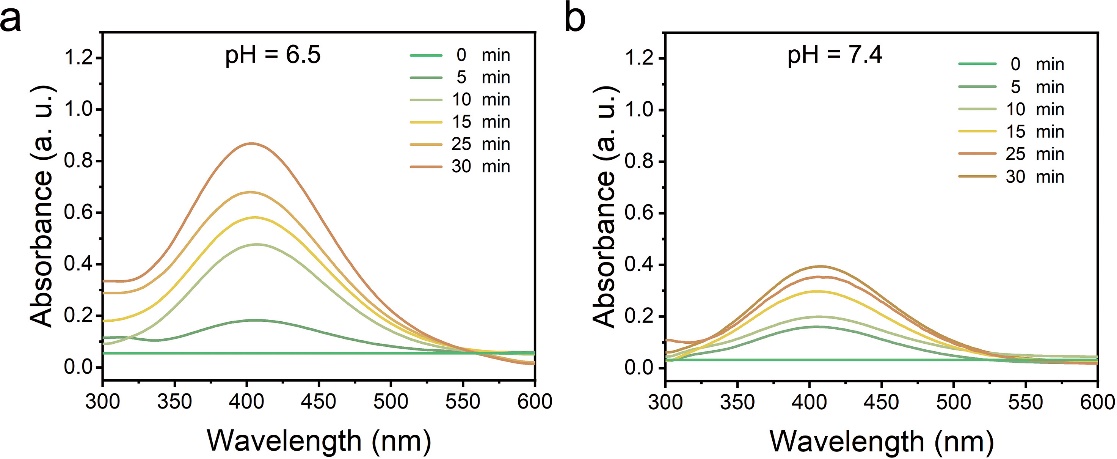


**Figure S9**. UV–vis absorption spectra of Ti(SO_4_)_2_ solution mixed with MgO_2_-CuO_2_@HA NCs solutions for different reaction times at (a) pH = 6.5 and (b) pH = 7.4.


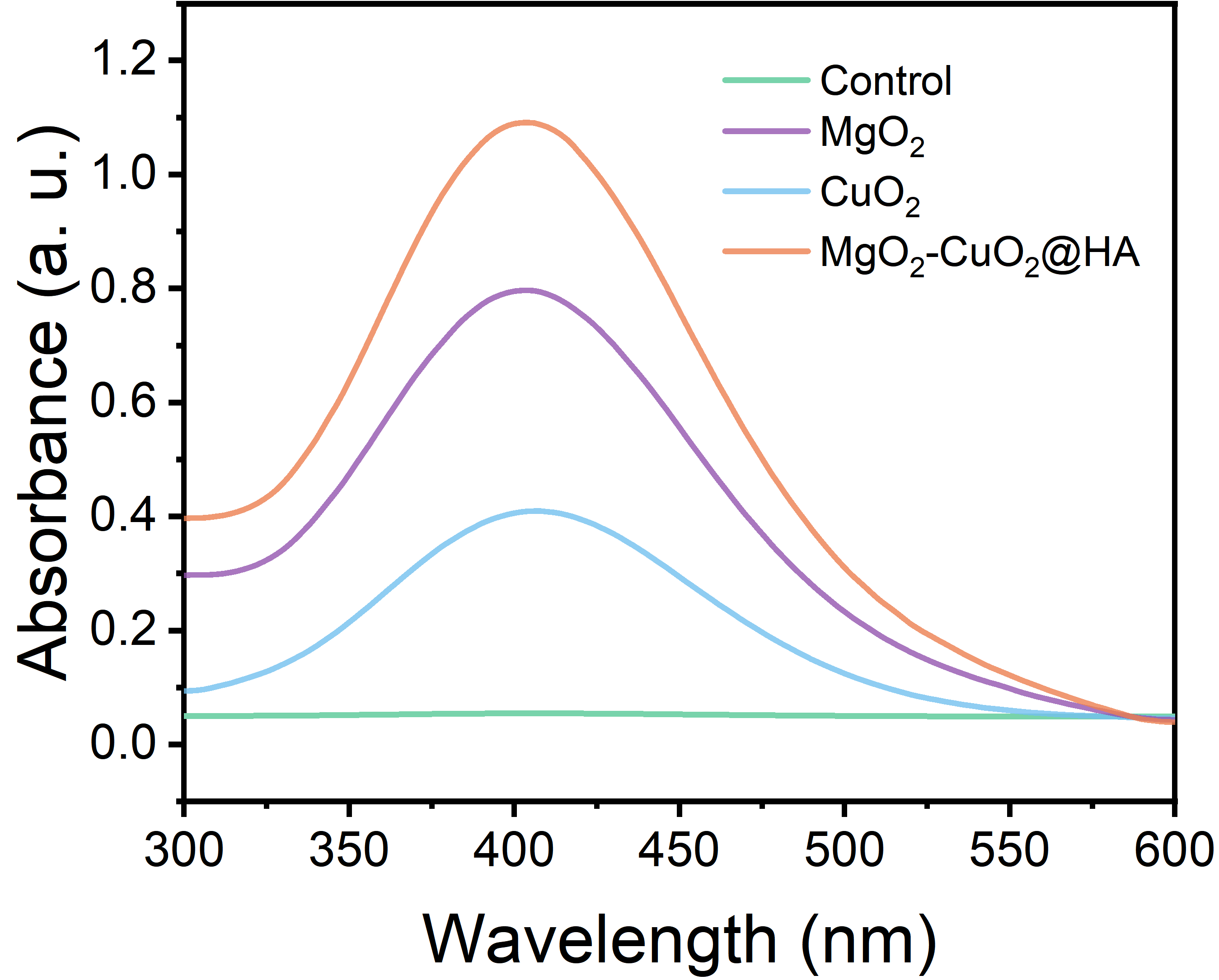


**Figure S10**. UV–vis absorption spectra of Ti(SO_4_)_2_ solution analyzed after 30 min of treatments with various groups at pH = 5.5, including Control, MgO_2_, CuO_2_, and MgO_2_-CuO_2_@HA, respectively.


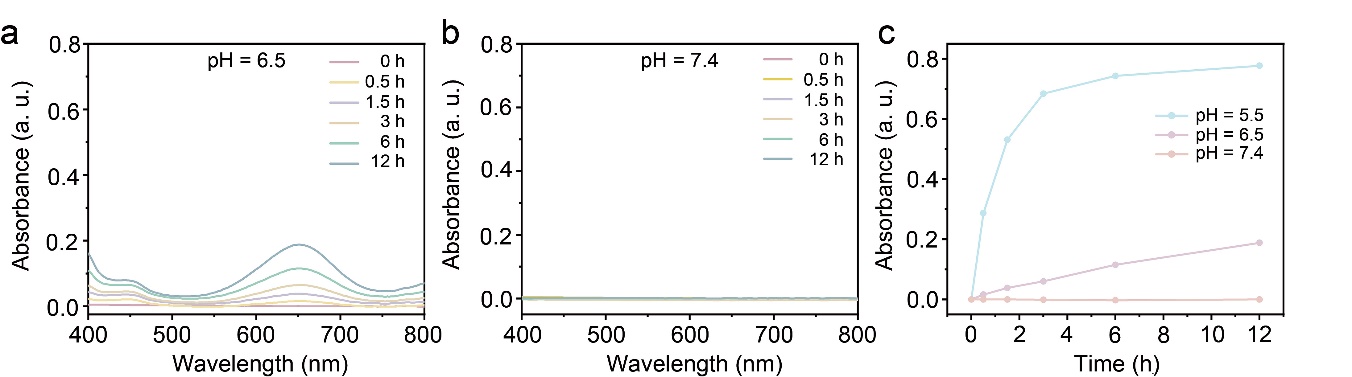


**Figure S11**. UV–vis absorption spectra of TMB solution mixed with MgO_2_-CuO_2_@HA NCs solutions at (a) pH = 6.5 and (b) pH = 7.4. (c) The quantitative analysis of absorbance changes at different experimental conditions.


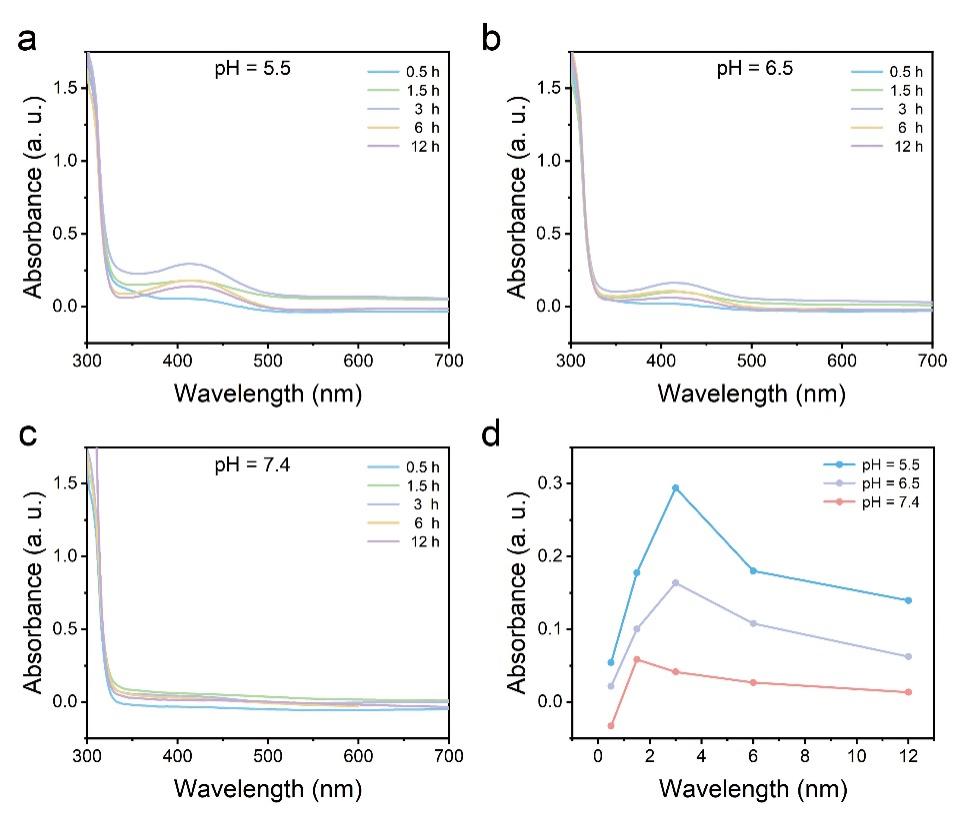


**Figure S12**. UV–vis absorption spectra of OPD solution mixed with MgO_2_-CuO_2_@HA NCs solutions at (a) pH = 5.5, (b) pH = 6.5, and (c) pH = 7.4, respectively. (d) The quantitative analysis of absorbance changes was performed under different conditions.


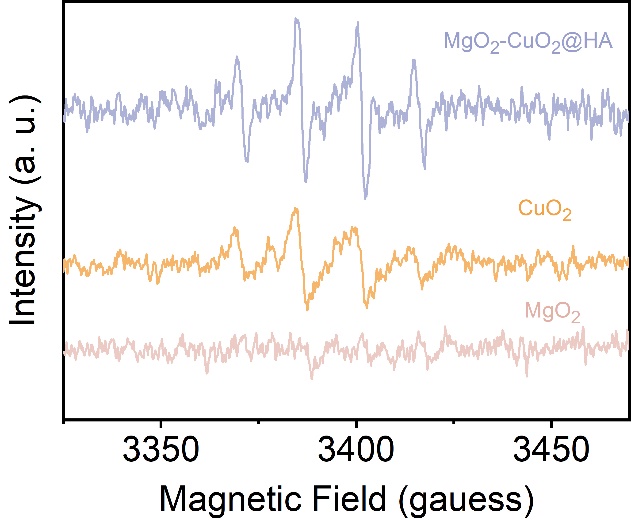


**Figure S13**. ESR spectra of DMPO-•OH adducts generated by MgO_2_, CuO_2_, and MgO_2_-CuO_2_@HA NCs after treatment with mild acid (pH = 5.5).


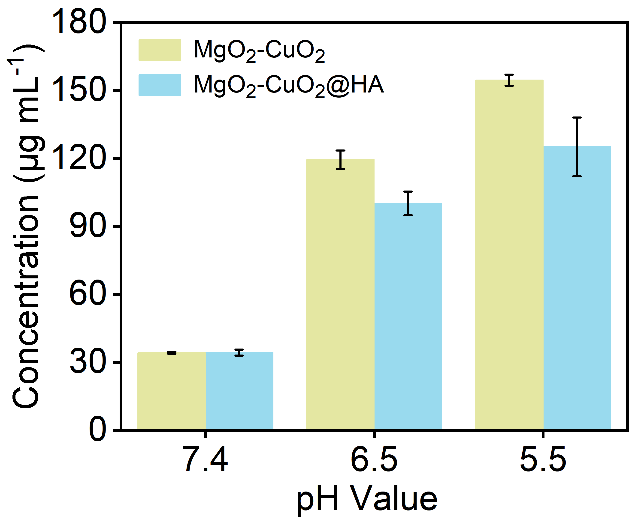


**Figure S14**. Mg^2+^ release from MgO_2_-CuO_2_ and MgO_2_-CuO_2_@HA NCs aqueous solutions with different pH values. Data are expressed as mean ± S.D. (n = 5).


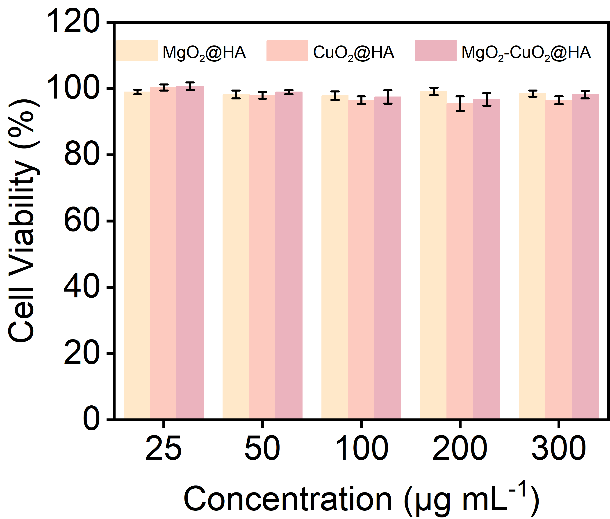


**Figure S15**. Cell viability of L929 cells with different treatments. Data are expressed as mean ± S.D. (n = 5).


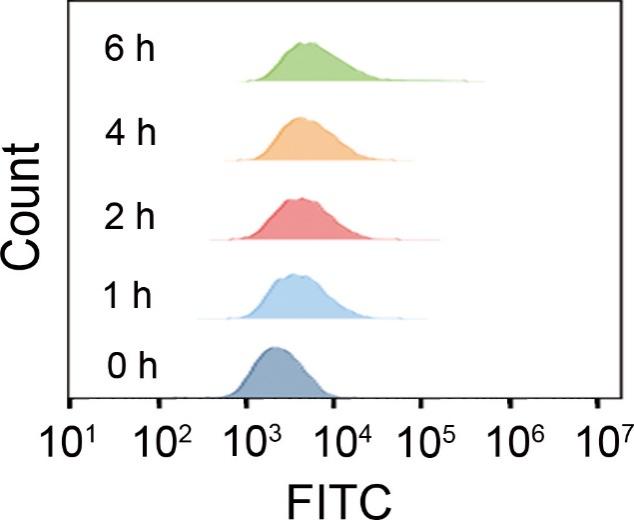


**Figure S16**. The time-dependent cellular uptake of FITC-modified MgO_2_-CuO_2_ NCs in 4T1 cells was quantified by flow cytometry.


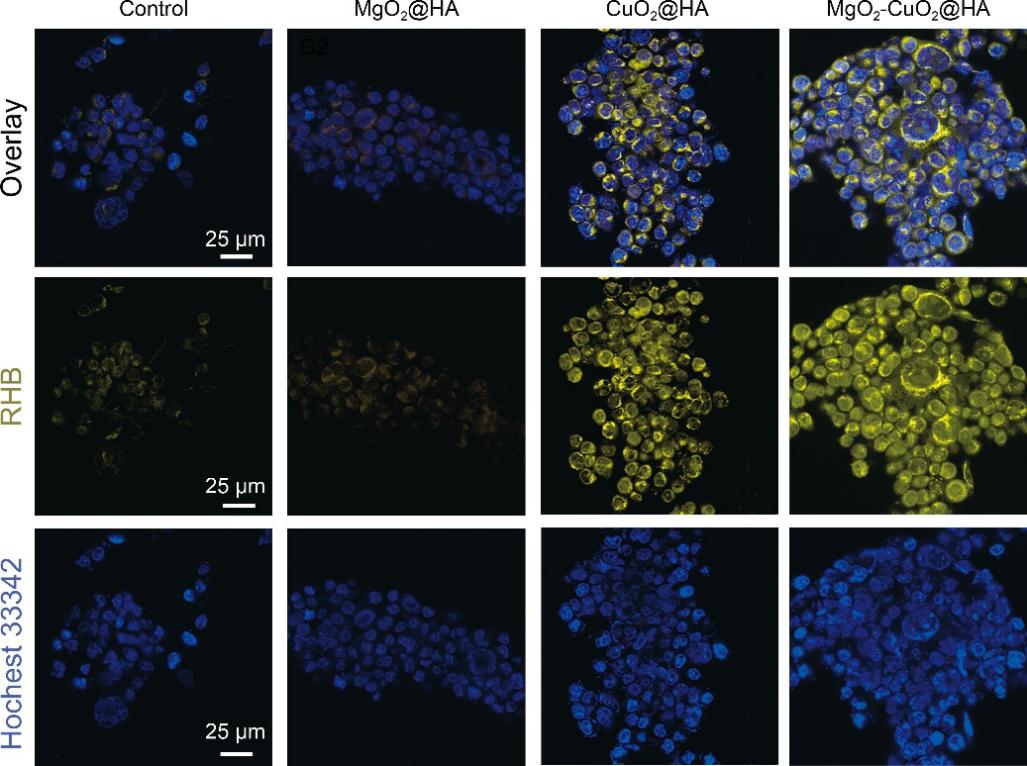


**Figure S17**. CLSM images of 4T1 cells stained with RBH after different treatments for 4 h.


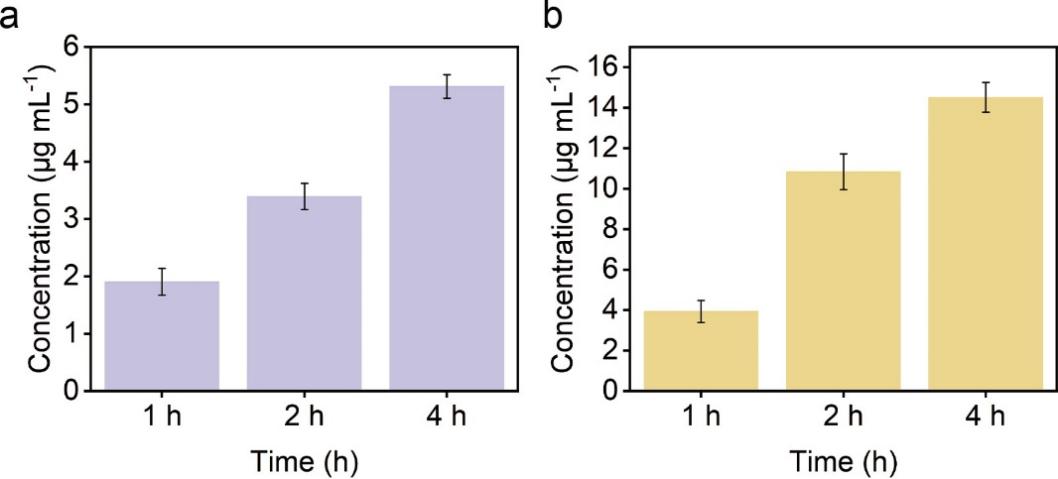


**Figure S18**. The Cu^2+^ concentration (a) and Mg^2+^ concentration (b) in 4T1 cells at different incubation times with MgO_2_-CuO_2_@HA NCs. Data are expressed as mean ± S.D. (n = 5).


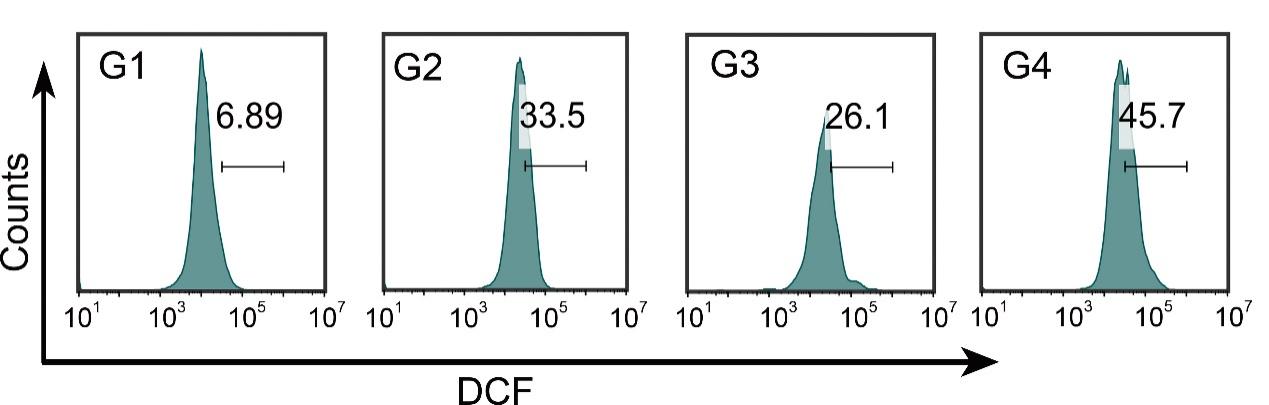


**Figure S19.** Flow cytometry profile of intracellular ROS levels after the 4T1 cells treated with different formulations including (G1) Control, (G2) MgO_2_@HA, (G3) CuO_2_@HA, and (G4) MgO_2_-CuO_2_@HA.


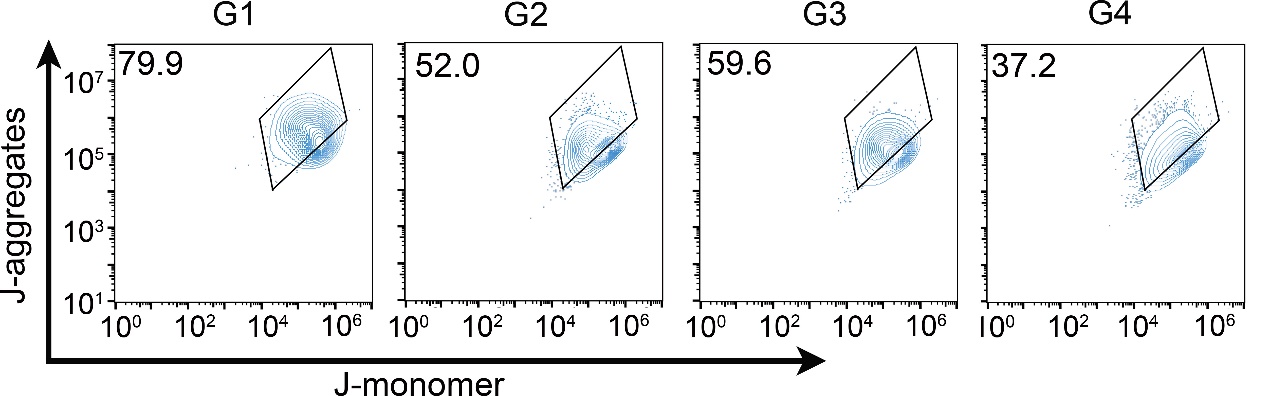


**Figure S20**. Flow cytometry profile of JC-1 after the 4T1 cells treated with different formulations including (G1) Control, (G2) MgO_2_@HA, (G3) CuO_2_@HA, and (G4) MgO_2_-CuO_2_@HA, respectively.


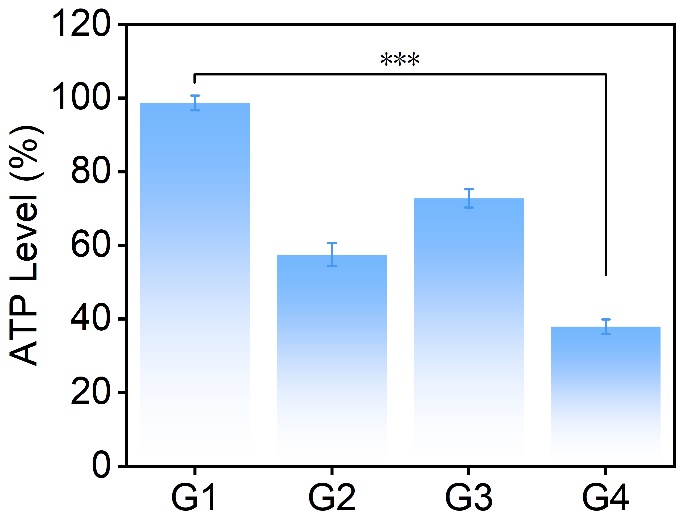


**Figure S21**. The measurement of intracellular ATP levels in different treatment groups, containing (G1) Control, (G2) MgO_2_@HA, (G3) CuO_2_@HA, and (G4) MgO_2_-CuO_2_@HA NCs. Data are expressed as mean ± S.D. (n = 5). Statistical significance was calculated using Student's *t*-test: ****p* < 0.001.


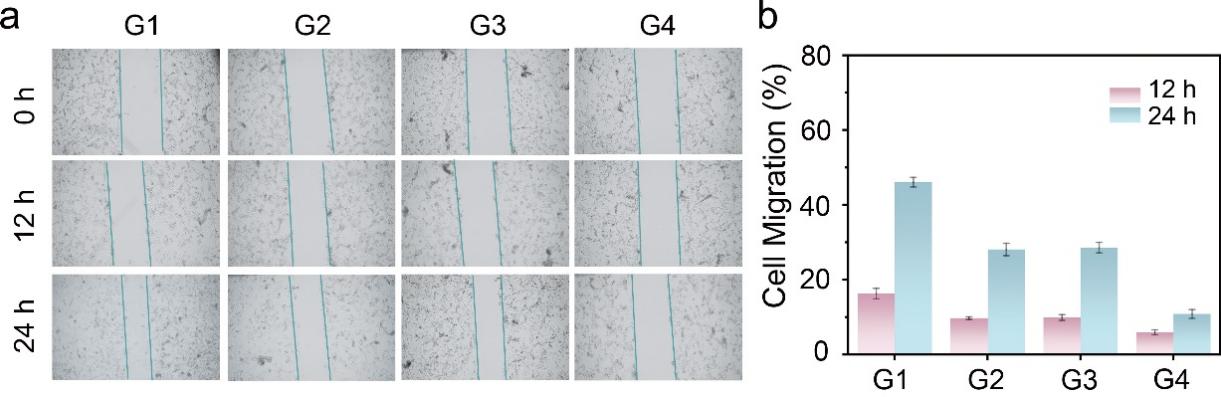


**Figure S22**. (a) Wound-healing assays and (b) the corresponding quantitative analysis of 4T1 cells treated with various formulations during the incubation process, the groups were set as (G1) Control, (G2) MgO_2_@HA, (G3) CuO_2_@HA, and (G4) MgO_2_-CuO_2_@HA. Data are expressed as mean ± S.D. (n = 5).


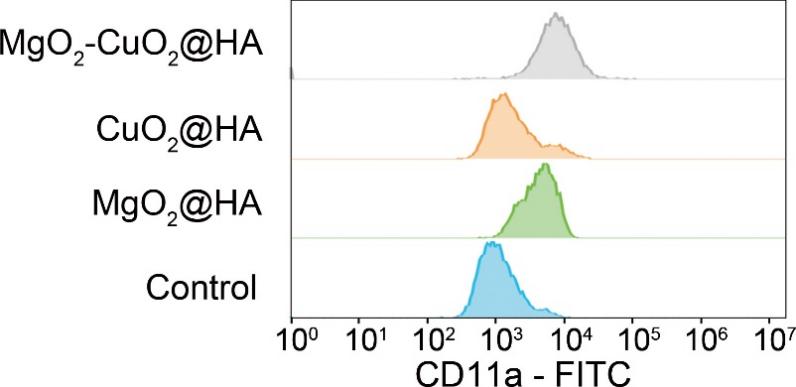


**Figure S23**. Flow cytometry analysis of LFA-1 expression on CD8^+^ T cells in different treatment groups.


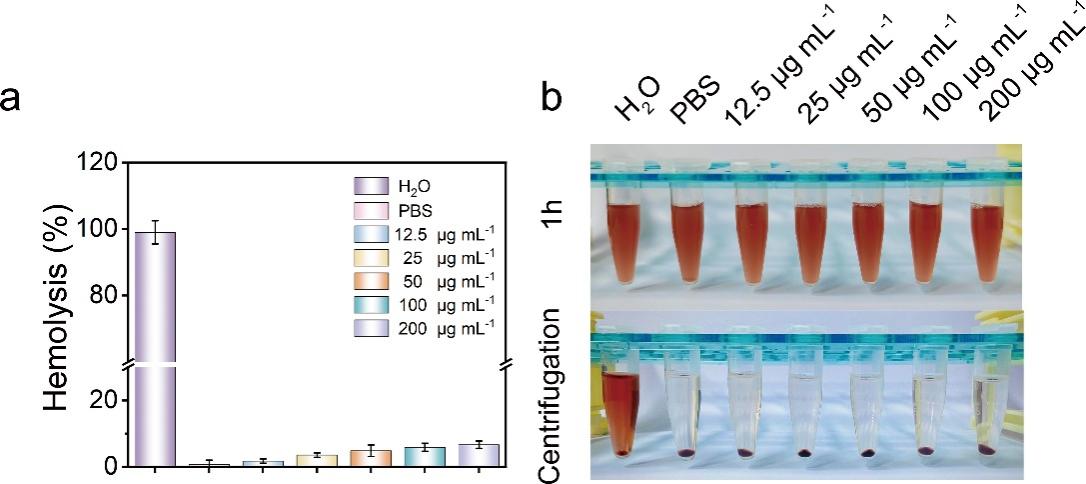


**Figure S24**. Hemolysis of red blood cells after incubation with MgO_2_-CuO_2_@HA NCs. (a) Hemolysis rate after incubation with different concentrations of MgO_2_-CuO_2_@HA NCs and (b) the corresponding digital photograph incubated with various concentrations of MgO_2_-CuO_2_@HA NCs, PBS (negative control), and water (positive control). Data are expressed as mean ± S.D. (n = 5).


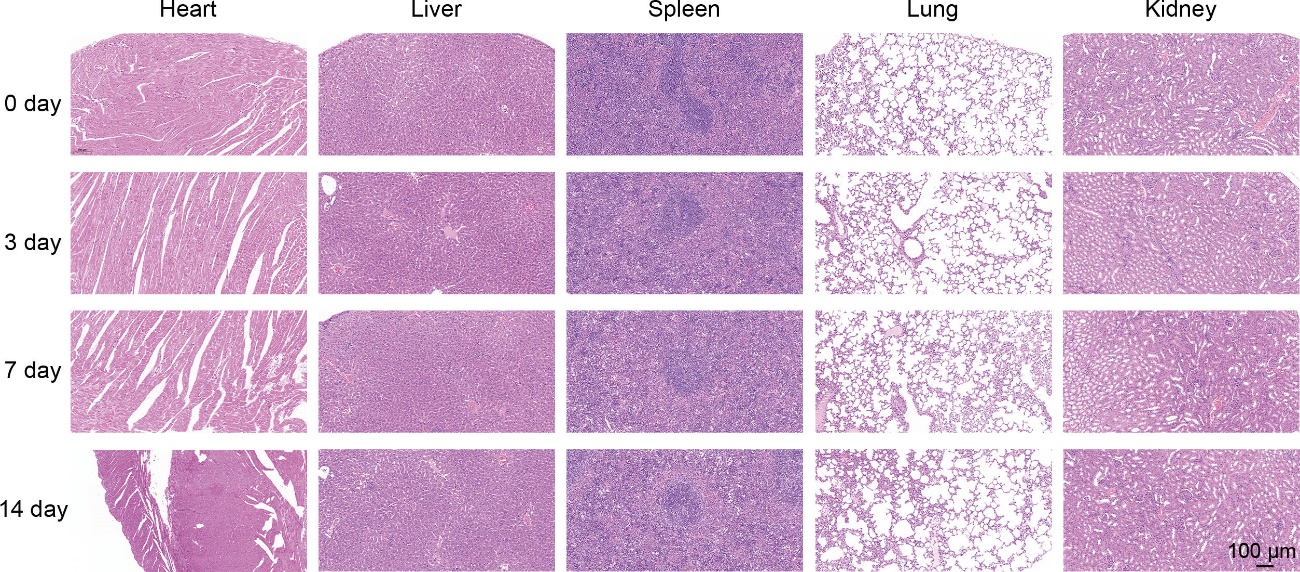


**Figure S25.** H&E staining images of heart, liver, spleen, lung, and kidney from the representative mice after administration of MgO_2_-CuO_2_@HA NCs for different time periods.


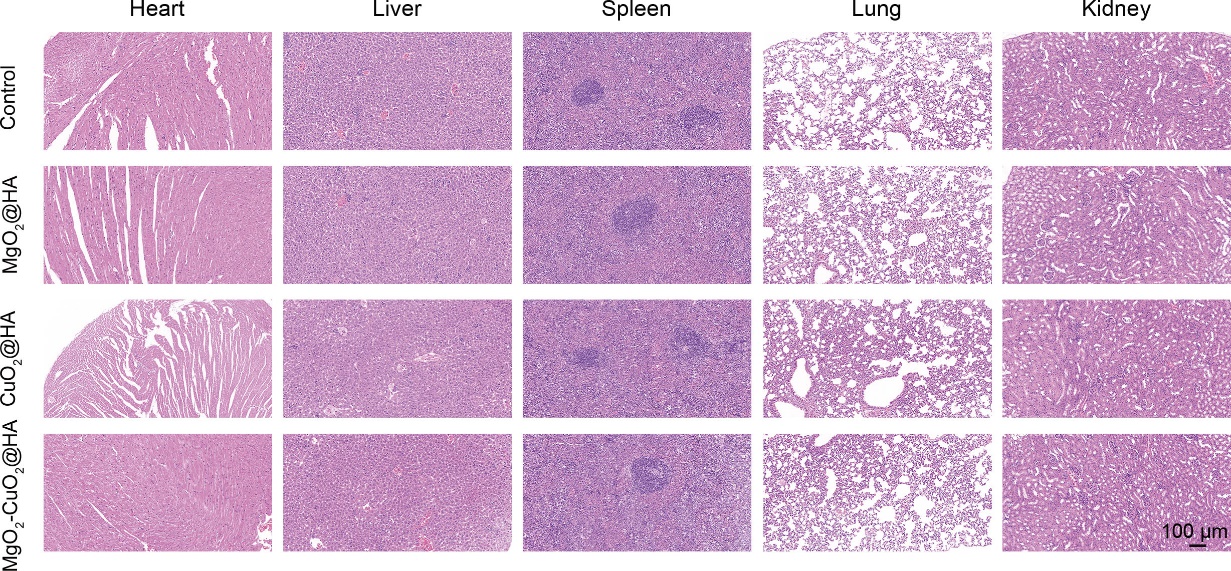


**Figure S26.** H&E staining images of heart, liver, spleen, lung, and kidney collected from the representative mice in different treatment groups.


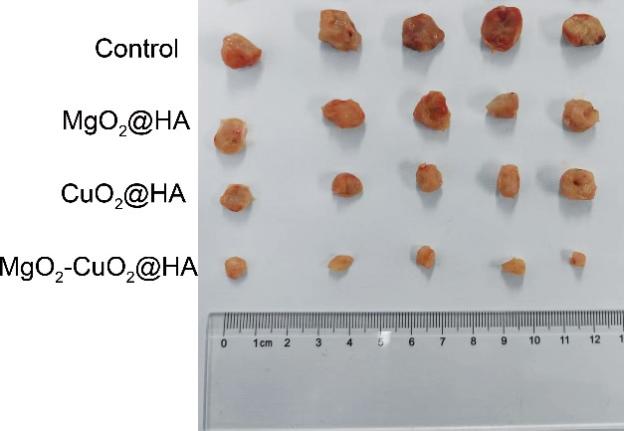


**Figure S27**. The corresponding photographs of excised tumors from different treatment groups.


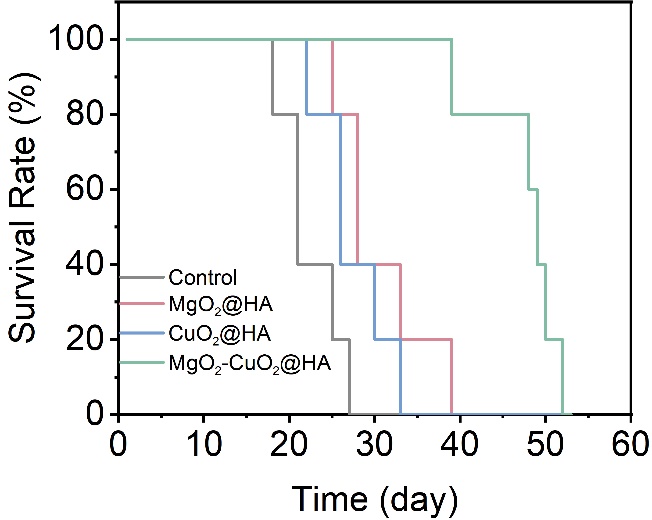


**Figure S28.** The survival rate curves of 4T1 tumor-bearing mice after treated with various formulations in a long-term monitoring.


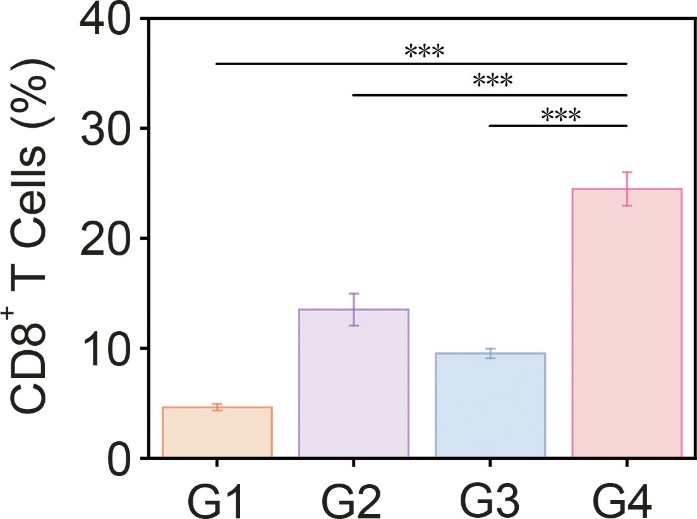


**Figure S29.** The semiquantitative analysis of CD8^+^T cells in different treatment groups including (G1) Control, (G2) MgO_2_@HA, (G3) CuO_2_@HA, and (G4) [MgO](mailto:MgO2-CuO2@HA).based)_[2](mailto:MgO2-CuO2@HA).based)_[-CuO](mailto:MgO2-CuO2@HA).based)_[2](mailto:MgO2-CuO2@HA).based)_[@HA based](mailto:MgO2-CuO2@HA).based) on the flow cytometry analysis result. Data are expressed as mean ± S.D. (n = 5). Statistical significance was calculated using Student's *t*-test: ****p* < 0.001.

**
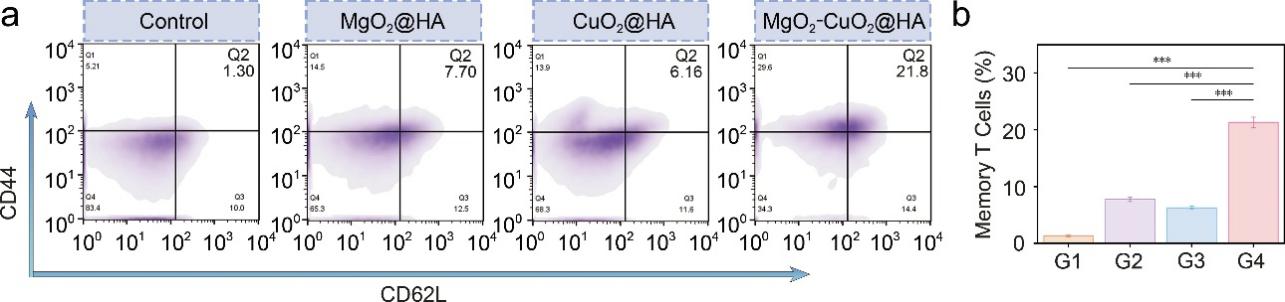
**

**Figure S30.** (a) Flow cytometry analysis of memory T cells in tumor tissue. (b) Quantitative analysis of memory T cells in tumor tissue in each treatment group, the groups were set as (G1) Control, (G2) MgO_2_@HA, (G3) CuO_2_@HA, and (G4) MgO_2_-CuO_2_@HA. Data are expressed as mean ± S.D. (n = 5). Statistical significance was calculated using Student's *t*-test: ****p* < 0.001.


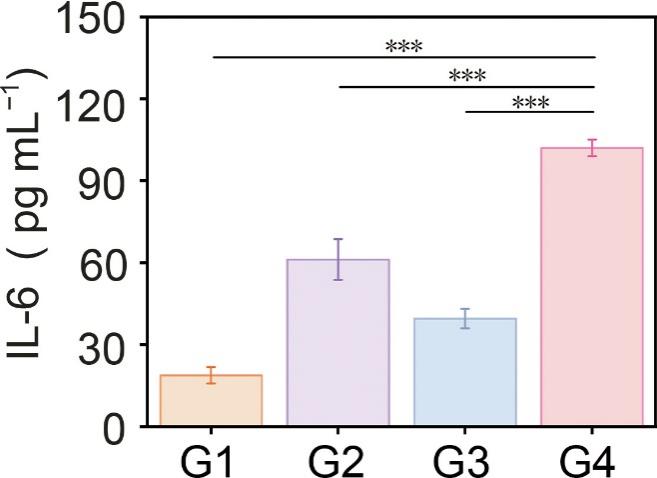


**Figure S31.** The levels of IL-6 cytokines in the serum of mice after various treatments were evaluated by ELISA assay, the groups were set as (G1) Control, (G2) MgO_2_@HA, (G3) CuO_2_@HA, and (G4) MgO_2_-CuO_2_@HA. Data are expressed as mean ± S.D. (n = 5). Statistical significance was calculated using Student's *t*-test: ****p* < 0.001.
